# Spatial and temporal variability of respiratory syncytial virus disease seasonality in Japan, 2012–2024

**DOI:** 10.1111/ped.70307

**Published:** 2025-12-27

**Authors:** Takeshi Arashiro, Ichiro Morioka, Naruhiko Ishiwada, Oliver Martyn, Rolf Kramer, Jing Jin, Amanda Pinho, Ewen Corbelon, Satoshi Kusuda

**Affiliations:** ^1^ Sanofi Vaccines Medical Tokyo Japan; ^2^ Graduate School of Medicine Chiba University Tokyo Japan; ^3^ Department of Pediatrics and Child Health Nihon University School of Medicine Tokyo Japan; ^4^ Department of Infectious Diseases, Medical Mycology Research Center Chiba University Chiba Japan; ^5^ Sanofi Vaccines Medical Lyon France; ^6^ Sanofi Vaccines Medical Beijing China; ^7^ HEOR, Real World Evidence IQVIA Solutions G.K Tokyo Japan; ^8^ Neonatal Research Network of Japan Kyorin University Tokyo Japan

**Keywords:** epidemiology, Japan, respiratory syncytial virus, seasonality, sentinel surveillance

## Abstract

**Background:**

Understanding infectious disease seasonality is crucial to inform immunization timing and risk communications. This study aimed to describe trends in respiratory syncytial virus (RSV) seasonality in Japan by determining thresholds across 47 prefectures using public health surveillance data.

**Methods:**

Weekly RSV cases per sentinel (CPS) is the main indicator used in Japan. Data from approximately 3000 pediatric sentinel sites were extracted for 2012–2024. Seasonal, advisory, and alert thresholds were established via two globally utilized methods (moving epidemic methods [MEM] and fixed threshold method). Based on the established seasonal threshold, onset/duration was examined for each season during the study period.

**Results:**

The fixed threshold method was finally chosen to establish the seasonal threshold, while medium and high‐intensity thresholds under the MEM methods were chosen as advisory and alert thresholds. The threshold values were 0.23–0.80CPS for seasonal, 0.75–2.82CPS for advisory, and 1.11–4.20CPS for alert. The epidemic periods usually lasted less than 6 months, with distinct epidemic peaks in almost all prefectures over the 13‐year observation. However, unlike other temperate countries/regions such as the United States and Europe, season onset shifted drastically from September–October in 2012/2013 to around March–April in 2024 with geographic variabilities. Calling season onset after 2 consecutive weeks above the threshold resulted in no false alarms in over half the prefectures.

**Conclusions:**

In countries such as Japan and the tropics/subtropics where the RSV seasons are less predictable, a flexible prevention strategy, tailored for each region/prefecture, using thresholds as guides, would ensure optimal protection against RSV and maximize public health benefits regardless of seasonal variability.

## BACKGROUND

Respiratory syncytial virus (RSV) is a highly contagious viral pathogen that can potentially cause severe disease in young children.^1^, ^2^ RSV infection typically exhibits distinct seasonality in temperate regions.^3^, ^4^ In Japan, RSV infection is monitored under the National Epidemiological Surveillance of Infectious Diseases (NESID) program. It is classified as a Category V infectious disease and reported by pediatric sentinel sites (“teiten”).^5^ Originally, a typical epidemic season occurred in the fall and winter before 2016, while substantial regional and temporal variations are thought to exist. The epidemic season shifted earlier into autumn during 2017–2019, and then into summer in 2020.^6^, ^7^ However, unlike seasonal influenza, which has a seasonal threshold of one reported case per sentinel site per week, there is no designated threshold for season onset by the government officially.^8^ Although several studies have attempted to determine RSV seasonal thresholds using the NESID data,^7^, ^9^, ^10^ these previous studies did not use statistical methods that are commonly utilized globally such as moving epidemic methods (MEM)^11^ and some were unable to estimate thresholds for most prefectures due to methodological limitations.^7^, ^10^ Also, there is no report that examined the duration of RSV season at the prefecture level utilizing globally accepted methods. Therefore, this study aimed to quantitatively describe recent trends in RSV seasonality by determining seasonal, advisory, and alert thresholds across 47 prefectures in Japan. Specifically, we assessed, the duration and onset of RSV seasonality both temporally since 2012 and spatially by prefecture.

## METHODS

### Data source

Under the NESID program, RSV cases in Japan are collected and published, and their occurrence and trends are assessed based on reports from physicians from sentinel sites nationwide. The sentinel sites are categorized as designated sentinel sites (~500 sites), pediatric sites (~3000 sites), influenza/COVID‐19 sites constituted of pediatrics/internal medicine (~5000 sites), and ophthalmology sites (~600 sites). Among those sentinel sites, only pediatric sites report RSV infection cases. Therefore, although reporting is not limited to children, NESID data reflect trends and levels of RSV epidemic among children.^12^ The reported cases have been confirmed based on either (1) detection of pathogens by isolation and identification using either nasal aspirates, nasal swabs, or throat swabs; (2) detection of pathogen antigens using rapid diagnostic kits; or (3) detection of antibodies in serum by neutralization reaction or complement fixation reaction. In Japan, RSV tests are covered by national health insurance (NHI) and most healthcare facilities use rapid antigen kits for testing. For RSV testing, any of the following groups is covered under NHI: (1) outpatient infants less than 1 year of age, (2) any hospitalized child, or (3) children eligible for palivizumab (note that the test is covered by NHI if any one of these criteria applies). To disseminate information acquired under the NESID Program, the Infectious Diseases Weekly Report (IDWR) is published every week by the National Institute of Infectious Diseases.^13^

### Indicators used

This study analyzed the data of weekly RSV cases per sentinel (CPS) from the IDWR. Since the data on “the number of CPS” was not available before week 9 in 2018, this study imputed the number of sentinel sites per prefecture based on the number of pediatric sentinels reported in the summary sheets (“shukei‐hyou”) in the NESID annual report^14^ for that period.

### Study design

This study was a retrospective observational study using publicly available aggregated data reported under the NESID program described in the previous section. The data period was from January 2012 through December 2024. Different time windows were used between the 2012/2013 and 2019/2020 seasons (from week 18 to week 17 of the following year) and the 2020 to 2024 seasons (from week 1 to week 52) due to the transition of the epidemic period in recent years (except for four prefectures in Kyushu [i.e., Nagasaki, Kumamoto, Miyazaki, and Kagoshima], where the cycle was defined as week 45 to week 44 of the following year after the 2020/2021 season, while in Kagoshima, the cycle was defined as week 18 to week 17 of the following year until the 2022/2023 season, and then week 1 to 52 thereafter).

The following approaches were employed: first, the epidemic curve was plotted to describe seasonality in recent years in Japan; second, several threshold candidates were considered at the prefecture level using the following methods: (i) MEM and (ii) RSV detections exceeding a fixed threshold of total annual RSV cases (hereafter “fixed threshold method”). These two methods are considered the most applicable among widely used methods identified in a previous systematic review,^11^ in terms of versatility and reproducibility with Japanese surveillance data. For the fixed threshold method, this study employed a 1.2% threshold of total annual RSV cases, which is most frequently used in previous studies.^11^, ^15^, ^16^, ^17^, ^18^ For MEM, it is possible to consider different levels of thresholds in addition to the seasonal threshold, so we explored these possibilities. Additionally, (iii) a sensitivity analysis using the method previously used in Japan using a relative operating characteristics (ROC) curve analysis was conducted for evaluating the stability and robustness of the two former methods.^7^ NESID data do not include the test positivity rate, so it was not feasible to set a threshold based on the positivity rate as done in some other countries including the United States.^19^ Data from 2021 were excluded in determining the thresholds due to extraordinarily intensive epidemic after the COVID‐19 pandemic.

Based on the exploration of the above three methods, the following three different thresholds were determined: (a) seasonal threshold; (b) advisory threshold; and (c) alert threshold. Among these, the seasonal threshold is the lowest, is expected to reflect the start and end of an epidemic period of RSV infections, and is the threshold that can be utilized to guide the start of prevention measures against RSV infections. The advisory and alert thresholds are expected to indicate RSV epidemics with moderate and high intensities, respectively.

The season onset and season duration were examined for each season during the study period using the established seasonal threshold. Prospectively, season onset may be misidentified by false alarms due to ups and downs in the beginning of the season after stable increases, especially when there are fewer sentinel sites with fewer reported cases. Therefore, some previous studies have used gap periods before declaring season onset.^20^, ^21^ In this study, 1‐ to 4‐week gaps were introduced in a 1.2% fixed threshold method, respectively, to infer the appropriate gap weeks to converge a single epidemic season for each prefecture. The appropriate gap weeks were evaluated for each prefecture based on the number of apparent epidemic seasons in each year.

Finally, annual cumulative CPS for each season was calculated using the weekly CPS data.

### Detailed statistical considerations on MEM, fixed threshold method, and ROC curve method

In MEM, the seasonal threshold (i.e., pre‐epidemic threshold) that marks the start of the epidemic period was established as the upper bound of the 95% one‐sided confidence interval of the geometric mean of the 30 highest pre‐epidemic weekly CPS (i.e., 30/number of seasons for each season) for over the data period.^22^ Medium and high intensity thresholds were determined based on the upper bounds of the 20% (medium intensity) and 40% (high intensity) one‐sided confidence intervals of the geometric mean of the 30 highest epidemic weekly CPS (i.e., 30/number of seasons for each season) for over the data period, respectively.^22^ In MEM, these thresholds reflect statistical boundaries used to classify medium and high‐intensity epidemic activities. In general, MEM is known to provide conservative estimates with low sensitivity and high specificity.^22^ In this study, these thresholds for RSV infection are assumed to reflect the level of strain on regional medical resources to accommodate pediatric inpatients.

In a 1.2% fixed threshold method, the epidemic start and end weeks were determined retrospectively based on the first and last weeks of the longest consecutive period in which the weekly CPS exceeded 1.2% of the total CPS for the year. In the base case, no gap week to prevent false alarms was introduced. Then, a single threshold over the study period for each prefecture was determined by the median of annual threshold values.

As a sensitivity analysis, the seasonal thresholds were compared with those estimated based on an ROC curve method.^7^ In this method, the epidemic period of RSV infection was defined by the time‐varying reproduction number (Rt), which enables determining the ascending phase of the epidemic with high sensitivity.^7^, ^23^

All statistical analyses and visualization were conducted using R software (R Core Team, 2024).

### Ethics approval, informed consent, data availability

This study was approved by Central Institutional Review Board (Shiba Palace Clinic, Tokyo, Japan; Approval number: 156979_rn‐38,728) on October 24 2024. Informed consent was waived for this study due to the use of deidentified aggregated public surveillance data. Data were extracted from the following URL: https://id‐info.jihs.go.jp/surveillance/idwr/jp/rapid/2025/40/index.html.

## RESULTS

### Establishing seasonal, advisory, and alert thresholds

**FIGURE 1:**
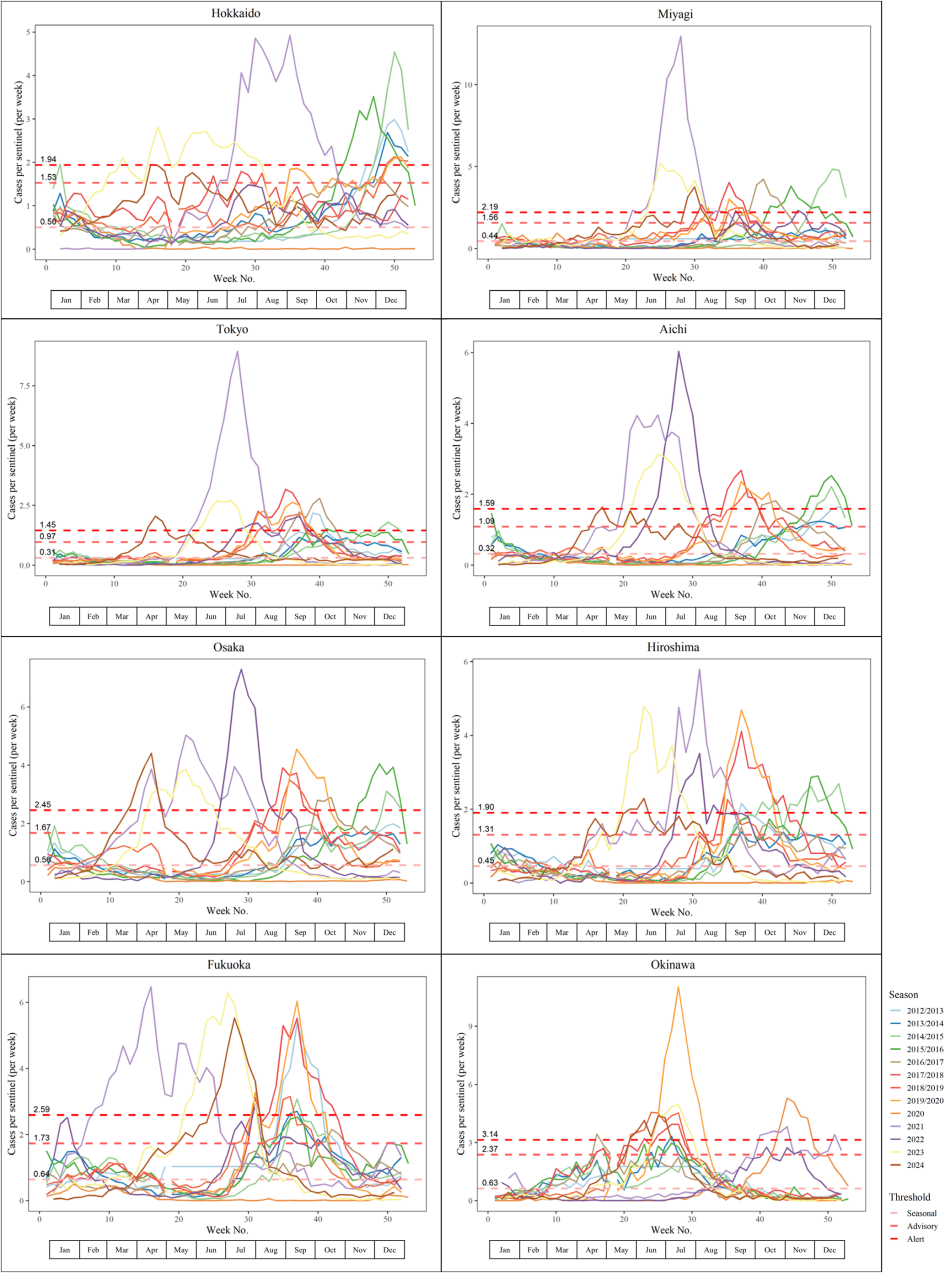
RSV epidemic curve with finalized thresholds nationwide and by prefecture in Japan, 2012–2024.

We first plotted epidemic curves nationwide as well as by prefecture. Most prefectures demonstrated distinctive epidemics/peaks with notable exceptions including Hokkaido and Nagasaki (Figure 1 and Figure S1). These exceptions and heterogeneity across prefectures could be due to differences in climate/latitude (similar to the variabilities observed globally depending on climate), in the distribution of sentinel sites (e.g., a sparse distribution or healthcare access can result in obscured seasonality), and urbanization or depopulation.^3^, ^4^

We next explored seasonal, advisory, alert threshold candidates and assessed their suitability in the context of Japanese surveillance data. With MEM, we set three different levels of threshold candidates (seasonal, medium intensity, and high intensity). The estimated threshold values were systematically highest among the three threshold methods. With the MEM seasonal threshold candidate, the values ranged from 0.31 to 1.70 CPS (Table S1; Figure S2). For medium intensity thresholds, the values range from 0.75 to 2.82 CPS, while for high‐intensity thresholds, they range from 1.11 to 4.20 CPS. With the 1.2% fixed threshold method, we set a seasonal threshold candidate. In contrast to MEM, this method presented with lower seasonal thresholds in general, ranging from 0.23 CPS (Kanagawa) to 0.80 CPS (Miyazaki). The results present similar geographical patterns with MEM (Pearson's correlation coefficients: 0.796). Finally, with the ROC method, which was one of the previously published methods in Japan, threshold values ranged from 0.17 CPS in Kyoto to 1.19 CPS in Niigata (Table S1). The ROC method resulted in lower correlations with the other methods: 0.596 versus MEM seasonal intensity, and 0.571 versus the 1.2% fixed threshold method based on Pearson's correlation coefficient, implying that the RSV activities in Japan are not always following a periodic probability function.

Based on the visual inspection of candidate thresholds and following a conservative approach to determine season onset as early as possible (since Japanese clinicians prefer to detect season onset as early as possible) to inform prophylaxis start timing or risk communication, together with the consistency with the previous estimates^10^ and the different method previously applied in Japan^7^ (Figure S2), we deemed that the 1.2% fixed threshold is more appropriate compared to the MEM seasonal threshold candidate in the context of Japanese surveillance data, and further analyses on season duration and onset were conducted using this as the seasonal threshold. For advisory and alert thresholds, MEM medium and high‐intensity thresholds were considered acceptable based on visual inspections (e.g., considering most seasons will experience intensity above advisory/alert thresholds), respectively (Table 1). Epidemic curves as well as the finalized threshold values were presented in Figure 1 and Figure S1. The threshold values ranged from 0.23 CPS (Kanagawa) to 0.80 CPS (Miyazaki) for the seasonal threshold, from 0.75 CPS (Kanagawa) to 2.82 CPS (Tokushima) for the advisory threshold, and from 1.11 (Kanagawa) to 4.20 CPS (Tokushima). Note that CPS varies significantly between prefectures, depending on factors such as population density and the urban–rural compositions (Figure S4).

**TABLE 1:** Finalized threshold values.

National/prefecture	Seasonal (based on 1.2% fixed threshold method)	Advisory (based on MEM medium [20%] threshold)	Alert (based on MEM high [40%] threshold)
Whole nation	0.418	1.178	1.683
Hokkaido	0.501	1.527	1.939
Tohoku			
Aomori	0.277	1.092	1.413
Iwate	0.396	1.593	1.994
Miyagi	0.439	1.557	2.194
Akita	0.254	0.937	1.378
Yamagata	0.617	2.399	3.531
Fukushima	0.648	2.016	2.864
Kanto			
Ibaraki	0.256	0.886	1.324
Tochigi	0.379	1.381	2.055
Gunma	0.300	1.271	1.848
Saitama	0.328	1.113	1.569
Chiba	0.240	0.792	1.147
Tokyo	0.308	0.966	1.447
Kanagawa	0.231	0.754	1.110
Chubu			
Niigata	0.624	2.008	2.863
Toyama	0.448	1.391	2.151
Ishikawa	0.427	1.574	2.389
Fukui	0.560	1.850	2.693
Yamanashi	0.232	0.926	1.437
Nagano	0.335	1.490	1.979
Gifu	0.344	1.067	1.654
Shizuoka	0.376	1.243	1.818
Aichi	0.317	1.085	1.589
Mie	0.548	1.848	2.788
Kinki			
Shiga	0.300	1.265	1.740
Kyoto	0.270	0.953	1.393
Osaka	0.558	1.668	2.453
Hyogo	0.456	1.474	2.165
Nara	0.514	1.668	2.546
Wakayama	0.483	1.766	2.318
Chugoku			
Tottori	0.489	1.625	2.619
Shimane	0.504	1.732	2.725
Okayama	0.285	1.045	1.544
Hiroshima	0.455	1.306	1.902
Yamaguchi	0.706	2.371	3.097
Shikoku			
Tokushima	0.784	2.824	4.195
Kagawa	0.516	1.976	2.918
Ehime	0.612	1.784	2.851
Kochi	0.470	1.816	2.547
Kyushu			
Fukuoka	0.636	1.731	2.587
Saga	0.550	1.627	2.568
Nagasaki^a^	0.478	1.193	1.978
Kumamoto^a^	0.550	1.583	2.463
Oita	0.478	1.450	2.276
Miyazaki^a^	0.796	1.861	3.293
Kagoshima^b^	0.651	2.168	2.858
Okinawa	0.626	2.372	3.136

^a^ Time window is from week 18 to week 17 of the following year by the 2019/2020 season, then from week 45 to week 44 of the following year thereafter.

^b^ Time window is from week 18 to week 17 of the following year by the 2022/2023 season, then from week 1 to 52 thereafter.

### Length of seasons by prefecture

**FIGURE 2:**
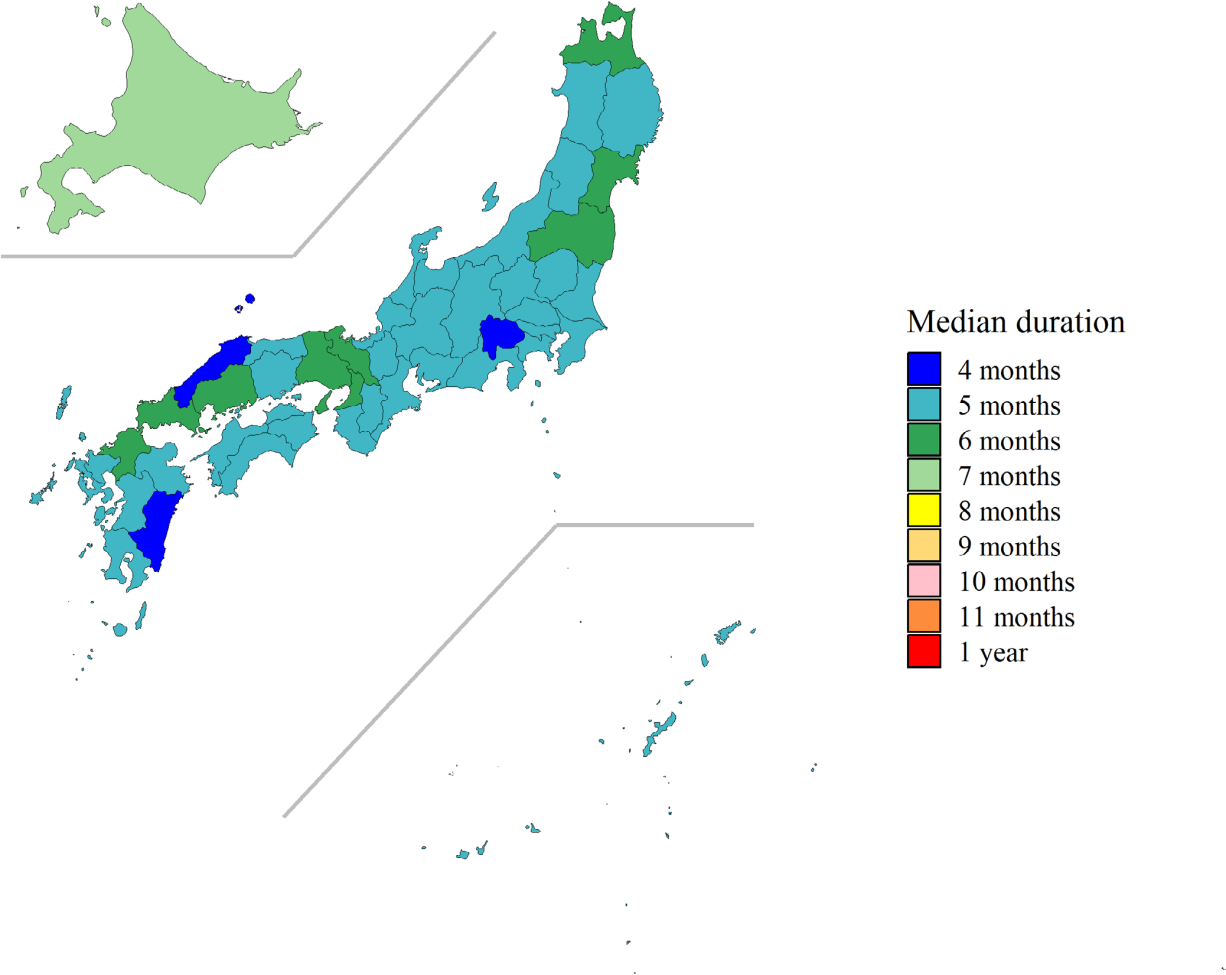
Map of Japan showing the median length of RSV epidemic seasons, 2012–2024. For Nagasaki, Kumamoto and Miyazaki, the time window was from week 45 to week 44 of the following year for the 2020/2021–2023/2024 seasons, while for Kagoshima, the time window was from week 18 to week 17 of the following year during the 2020/2021–2022/2023 seasons, then week 1–52 thereafter.

**FIGURE 3:**
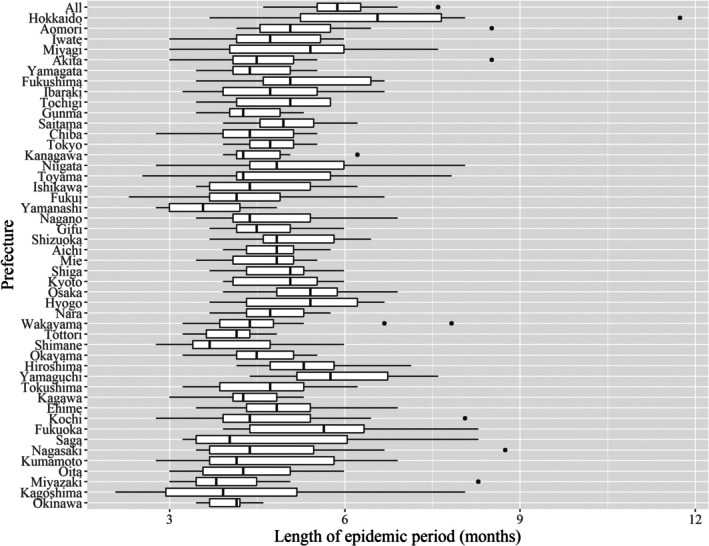
Spatial and temporal variabilities of the durations of RSV epidemic periods in Japan, 2012–2024. In the figure, the left and right sides of the box represent the 25th and 75th percentiles, respectively. The vertical line inside the box represents the median. Whiskers extend to the most extreme data points not considered outliers (i.e., greater than 1.5 times the interquartile range from the 25th and 75th percentiles, respectively), and the dots represent outliers. For Nagasaki, Kumamoto and Miyazaki, the time window was from week 45 to week 44 of the following year for the 2020/2021–2023/2024 seasons, while for Kagoshima, the time window was from week 18 to week 17 of the following year during the 2020/2021–2022/2023 seasons, then week 1–52 thereafter.

Using the seasonal threshold determined via the 1.2% fixed threshold, we estimated the length of seasons by prefecture for the past 13 years. In 37 of the 47 prefectures, except for Hokkaido, Aomori, Miyagi, Fukushima, Kyoto, Osaka, Hyogo, Hiroshima, Yamaguchi, and Fukuoka, the median epidemic periods were within 5 months (i.e., by 21 weeks), with the minimum median epidemic period in Yamanashi (3.5 months or 15 weeks) (Figures 2, 3; Table S2; Figure S3). Hokkaido exhibited the longest epidemic period among all prefectures, with a median duration of 6.5 months (28 weeks). In almost all prefectures except for Hokkaido, the median epidemic periods were within 6 months.

### Season onset by prefecture

**FIGURE 4:**
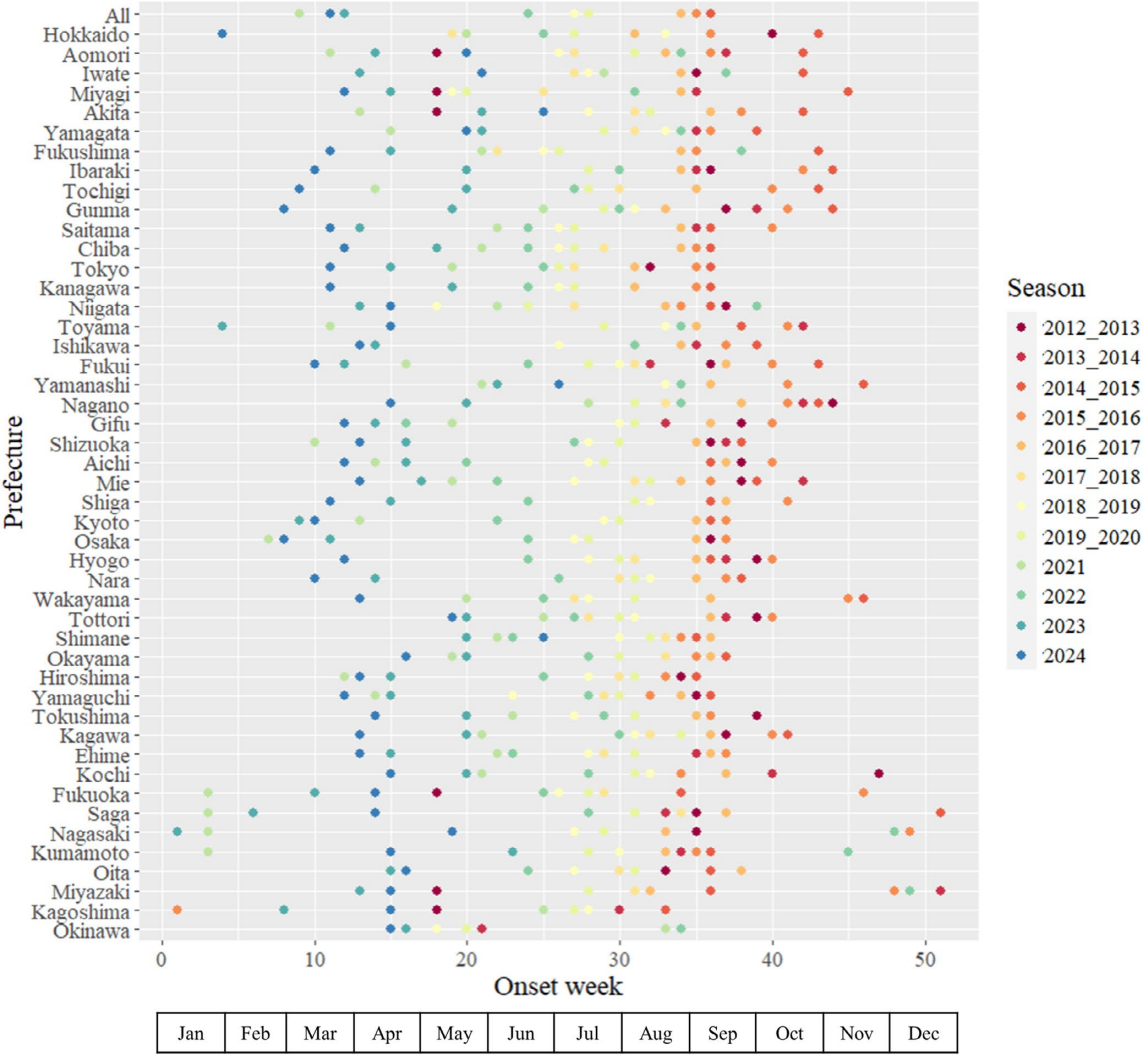
Spatial and temporal patterns of RSV epidemic onset in Japan, 2012–2024. For Nagasaki, Kumamoto and Miyazaki, the time window was from week 45 to week 44 of the following year for the 2020/2021–2023/2024 seasons, while for Kagoshima, the time window was from week 18 to week 17 of the following year during the 2020/2021–2022/2023 seasons, then week 1–52 thereafter.

We next examined the season onset by prefecture for each year. There were wide geographic variabilities. The season onsets were around September–October in the beginning of the study period in the 2012/2013 season but shifted earlier even before the pandemic with the onset between March and April in the latest year (2024 season) (Figure 4; Table S3).

### Specificity of seasonal threshold for prospective use

With no gap period, a median of one epidemic was observed per year in 9 out of 47 prefectures (Table S4). However, when a 1‐week gap period was introduced, the median number of apparent epidemic seasons per year converged to one in more than half (29) of the 47 prefectures. In 38 of the 47 prefectures, the median number of apparent epidemic seasons during the study period converged to one when a 2‐week gap period was used. On the other hand, in several prefectures (Aomori, Akita, Gunma, Fukui, Yamanashi, Nara, Ehime, Kagoshima, and Okinawa), a longer gap period would be needed.

### Cumulative CPS per year

Finally, we examined the annual cumulative CPS with a hypothesis that there would be similar cumulative cases every year regardless of the variability in the pattern of the epidemic curve. Outside of extremely low cumulative CPS in the 2020 season and extremely high cumulative CPS in the 2021 season which is likely associated with the COVID‐19 pandemic, the annual cumulative CPS was similar over time albeit with a gradual increase in cumulative CPS across the study period that were observed in most of the prefectures (Table S5 and Figure S4).

## DISCUSSION

This study described trends in the onset and duration of RSV seasonality in Japan from 2012 to 2024 by setting seasonal, advisory, and alert thresholds across 47 prefectures using sentinel surveillance data in Japan. Unlike the previous studies conducted in Japan, this study estimated epidemic periods with methods commonly used globally including MEM and 1.2% fixed threshold method.^11^, ^15^

First, epidemic curves plotted by prefecture showed distinctive epidemics/peaks for most prefectures. As observed in other countries, there was a suppression of the epidemic in 2020 and a large surge in 2021, likely due to the COVID‐19 pandemic altering preventive behaviors and a possible change in the intensity of surveillance.

When exploring seasonal threshold candidates with different methods, MEM generally showed higher threshold values (i.e., more conservative threshold with lower sensitivity and higher specificity in terms of calling the season onset) (Figure S2). This is in line with previous studies providing higher thresholds with high specificity.^22^ Meanwhile, the 1.2% fixed threshold method resulted in lower threshold values (i.e., less conservative threshold with lower specificity but higher sensitivity in terms of determining the season onset). Based on visual inspection and following a conservative approach to determine season onset as early as possible (since Japanese clinicians prefer to detect season onset as early as possible) to inform prophylaxis start timing or risk communication, we deemed that the 1.2% fixed threshold is more appropriate, and further analyses were conducted using this as the seasonal threshold (Figure 1 and Figure S1). We also explored the specificity of the seasonal threshold for prospective use. Despite its low specificity inherent to the method, the seasonal threshold with the 1.2% threshold method resulted in no false alarm on average in more than half of the prefectures with the introduction of a 1‐week gap period, meaning that calling season onset after 2 consecutive weeks above threshold resulted in no false alarms in over half the prefectures (Table S4). This combination (1.2% threshold method with 1‐week gap) may be the most suitable for clinical practice, where early identification of the onset of the epidemic season is particularly important. However, it is important to note that NESID data are delayed by 1–2 week(s) and the use of local surveillance data (which is generally available a week earlier compared to NESID) as well as the use of multiple sources of information in decision‐making are warranted.

When we examined the length of seasons by prefecture, the median epidemic period was within 6 months for most prefectures, despite the fact that we decided to adopt the method that would result in longer seasonality due to generally lower threshold values (Figure 1). These were in line with or slightly longer compared to other countries in the temperate region,^4^, ^24^ although this could be due to the choice of a less conservative seasonal threshold. The season onset shifted drastically over the study period from September–October at the beginning of the study period in the 2012/2013 season to March–April in the 2024 season with wide geographic variabilities. This trend is in stark contrast to what is observed in other countries in the temperate region,^19^, ^25^ and is more similar to patterns seen in tropical regions, which are characterized by longer, and more variable activity.^26^ In countries/regions such as the United States and Europe, the disruption in the RSV seasonality was only temporarily observed in 2021 and recovered back to regular from fall to winter season. Climate change could be one factor,^27^ but further investigation is needed to understand the factors that caused this unique seasonality in Japan in recent years. This is in contrast to what is observed for influenza seasonality in Japan, where the seasonality has been consistent in the wintertime except during the COVID‐19 pandemic.^27^ However, in the meantime, these results reiterate the importance of continuous monitoring via routine public health surveillance to inform risk communication and decision‐making.

Our results also have implications for the implementation of emerging prevention measures against RSV infections, including maternal immunization and the long‐acting monoclonal antibody, nirsevimab, for infants. For now, clinical data on the duration of protection is up to 5–6 months for both products, although some data are available to suggest a longer duration of protection for nirsevimab.^28^, ^29^, ^30^, ^31^ Our results with distinctive epidemics of up to 6 months for most prefectures (with some exceptions such as Hokkaido), but with variable onset pose a challenge in when to administer these new preventive measures. However, either way, a flexible prevention approach that is tailored for each region/prefecture to maximize public health benefits is warranted.

The study will also inform how to consider RSV seasonality in countries such as those in the tropics and subtropics regions where the seasonality is not as apparent as in the United States and Europe.^4^, ^32^

## LIMITATIONS

There were several limitations of this study. First, detailed demographic characteristics of the study population were not available in IDWR, although, as mentioned in the Methods section, most cases reported are considered to be pediatric cases. Second, the final determination of the thresholds was done via visual inspection of candidate thresholds, although this is routinely done in other similar overseas studies to set thresholds.^11^, ^15^, ^16^, ^17^, ^18^ It is important to note that threshold determination is a tradeoff of sensitivity and specificity and local contexts should be taken into account in determining the optimal method. Third, the MEM model may cause bias when there are two or more epidemic outbreaks within the same time window,^23^ which are observed in some prefectures. Finally, there is an ongoing discussion by the national government to adjust the number of sentinel sites. After this change, it may be necessary to monitor whether the thresholds set in this study are appropriate. Nevertheless, the seasonality described during the study period (2012–2024) would still be valuable for public health and medical decision‐making.

## CONCLUSIONS

This study established epidemic thresholds of RSV with different intensities and examined regional and temporal variabilities of RSV seasonality using sentinel surveillance data in Japan. Using widely accepted MEM and the 1.2% fixed threshold method, seasonal, advisory, and alert thresholds were established. The epidemic periods usually lasted within 6 months over the 13‐year observation in almost all prefectures. The onset of the epidemic season shifted drastically from September–October (fall) in the 2012/2013 season to around March–April (spring) in 2024 with wide geographic variabilities. These results reiterate the importance of continuous monitoring. In countries such as Japan, where the RSV seasons are less predictable, a flexible prevention strategy is important. An adaptive immunization strategy approach, tailored for each region/prefecture, using thresholds as guides, would ensure optimal protection against RSV and maximize public health benefits regardless of seasonal variability.

## AUTHOR CONTRIBUTIONS

TA contributed to conceptualization, formal analysis, investigation, methodology, and writing—original draft; IM contributed to formal analysis, methodology, and writing—review and editing; NI contributed to formal analysis, methodology, and writing—review and editing. OM contributed to formal analysis, funding acquisition, methodology, and writing—review and editing; RK contributed to conceptualization, funding acquisition, methodology, and writing—review and editing; JJ contributed to conceptualization, methodology, and writing—review and editing; AP contributed to conceptualization, methodology, and writing—review and editing; EC contributed to formal analysis, methodology, and writing—review and editing; KS contributed to conceptualization, methodology, investigation, and writing—review and editing. All authors gave approval of the final version.

## FUNDING INFORMATION

The study was funded by Sanofi and AstraZeneca.

## CONFLICT OF INTEREST STATEMENT

Takeshi Arashiro is a Sanofi employee and a cooperative researcher at the National Institute of Infectious Diseases, Japan. Martyn Oliver, Rolf Kramer, Jing Jin, and Pinho Amanda are employees of Sanofi. Ewen Corbelon is an employee of IQVIA.

## Supporting information


**Figure S1.** RSV Epidemic curve with finalized thresholds nationwide and by prefecture in Japan, 2012–2024.

**Figure S2.** MEM with their thresholds.

**Figure S3.** Map of Japan showing the minimum and maximum lengths of the epidemic seasons.

**Figure S4.** Cumulative cases per sentinel.

Appendix S1.

## Data Availability

Research data is not shared.
