# Supplementary figures and images for: Spatial and temporal variability of respiratory syncytial virus disease seasonality in Japan, 2012–2024
Source: Pediatr Int. 2025 Dec 27;68(1):e70307. doi: 10.1111/ped.70307 (PMC12743260; doi:10.1111/ped.70307)

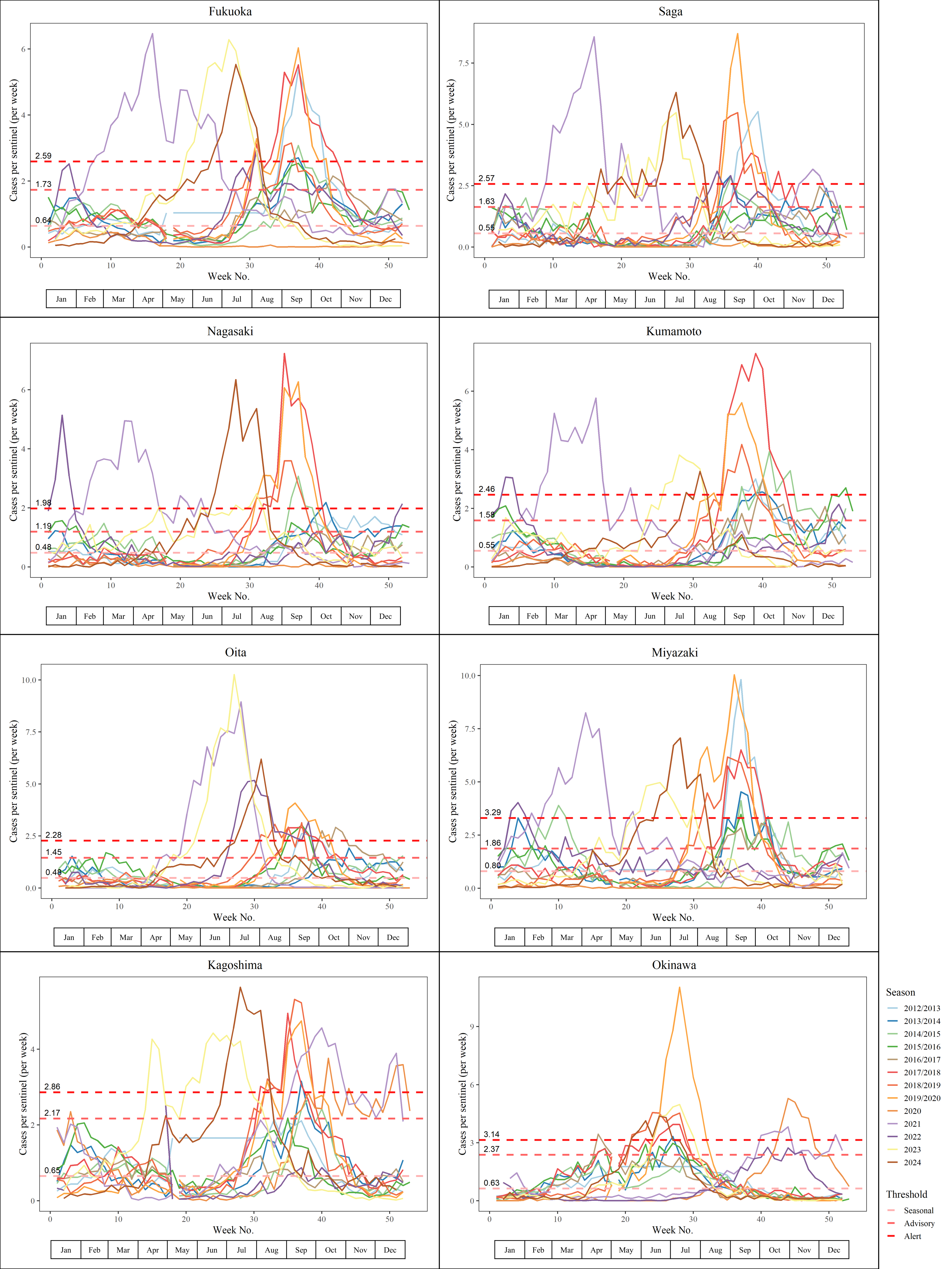

Supplement: Supplementary file 1 — Figure S1. RSV Epidemic curve with finalized thresholds nationwide and by prefecture in Japan, 2012–2024. [file PED-68-e70307-s004.zip › ped70307-sup-0006-FigureS1-S6@Supp_Figure1_6_highres.tif]

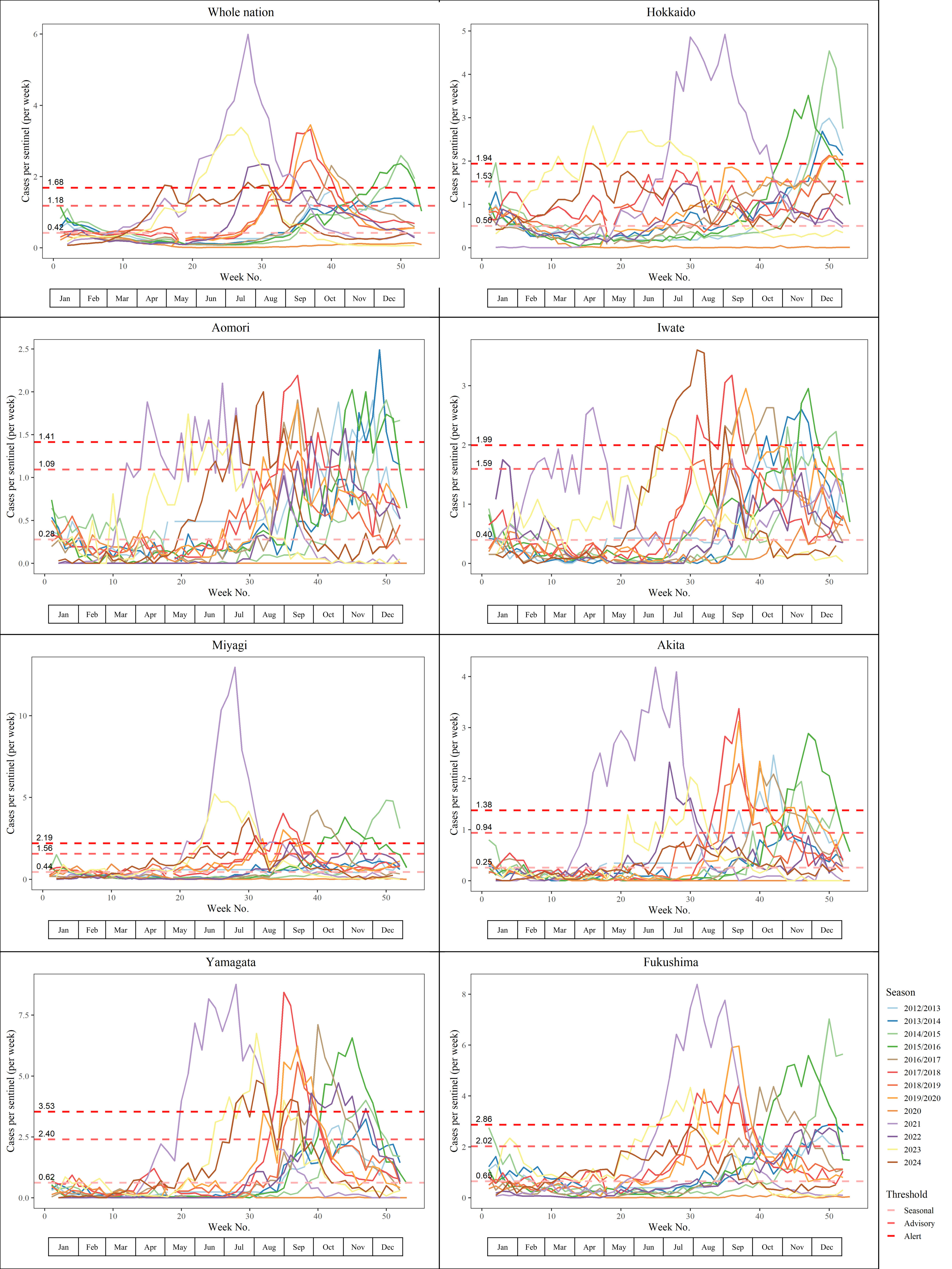

Supplement: Supplementary file 1 — Figure S1. RSV Epidemic curve with finalized thresholds nationwide and by prefecture in Japan, 2012–2024. [file PED-68-e70307-s004.zip › ped70307-sup-0001-FigureS1.tif]

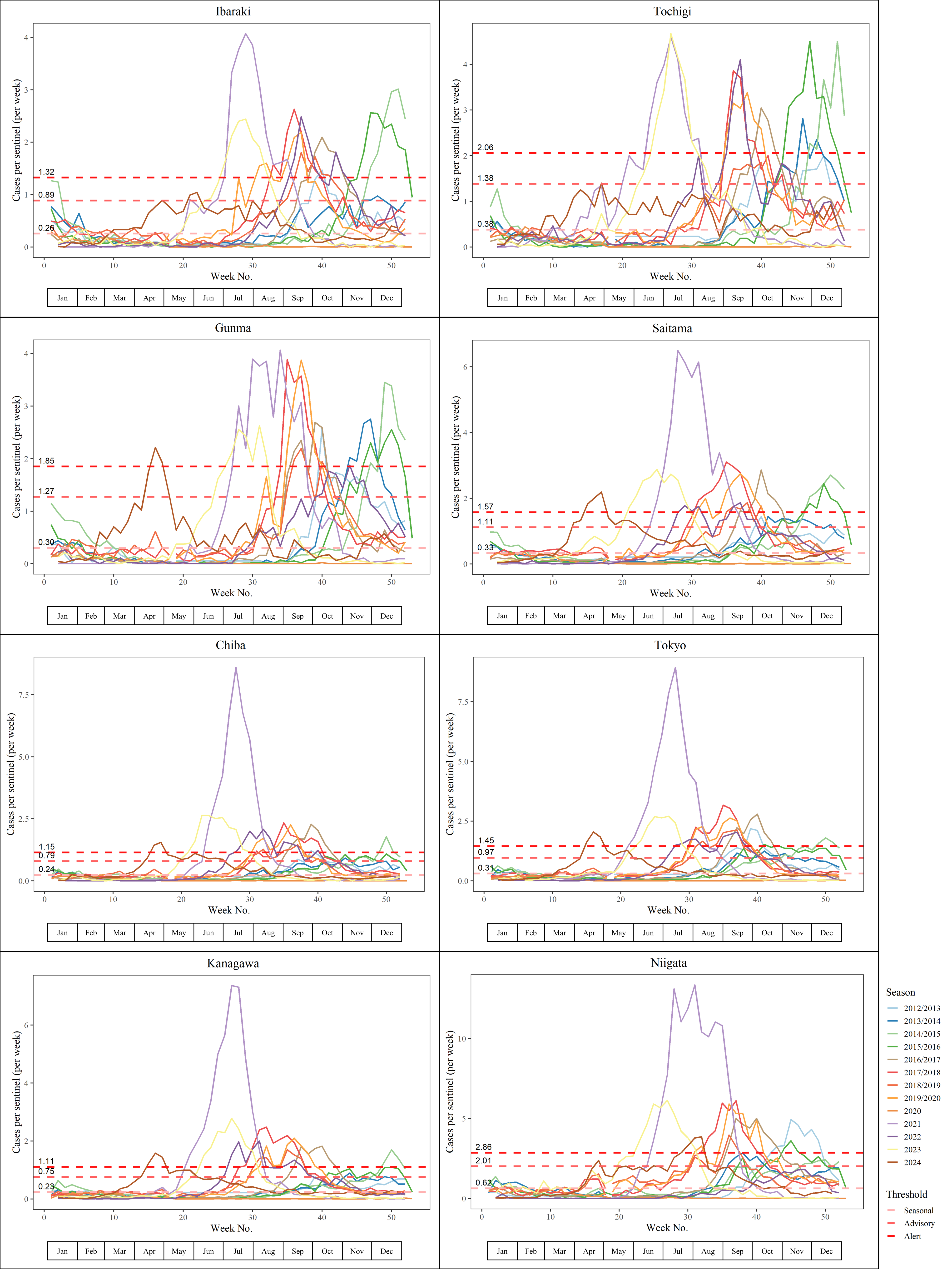

Supplement: Supplementary file 1 — Figure S1. RSV Epidemic curve with finalized thresholds nationwide and by prefecture in Japan, 2012–2024. [file PED-68-e70307-s004.zip › ped70307-sup-0002-FigureS1-S2@Supp_Figure1_2_highres.tif]

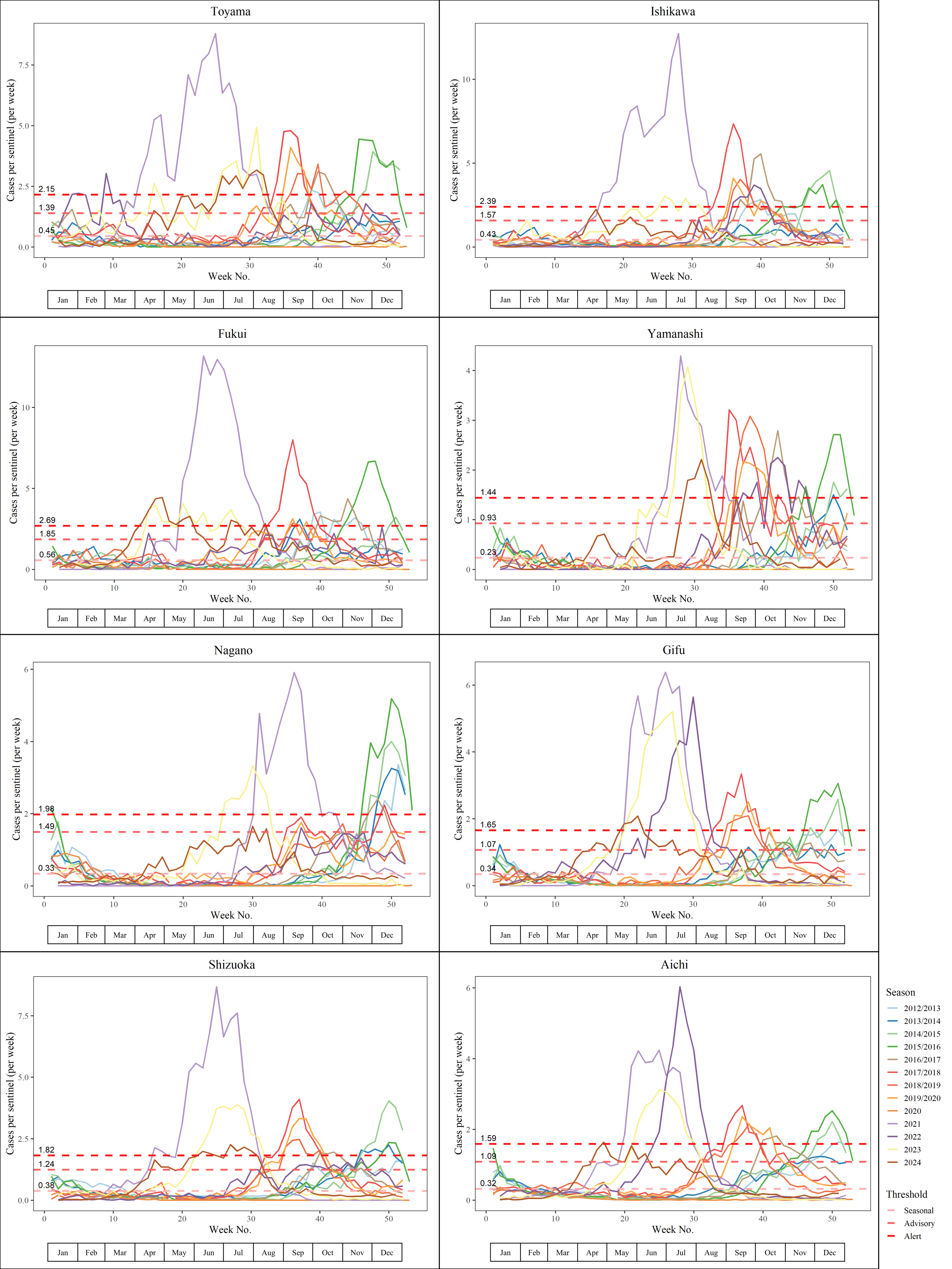

Supplement: Supplementary file 1 — Figure S1. RSV Epidemic curve with finalized thresholds nationwide and by prefecture in Japan, 2012–2024. [file PED-68-e70307-s004.zip › ped70307-sup-0003-FigureS1-S3@Supp_Figure1_3_highres.tif]

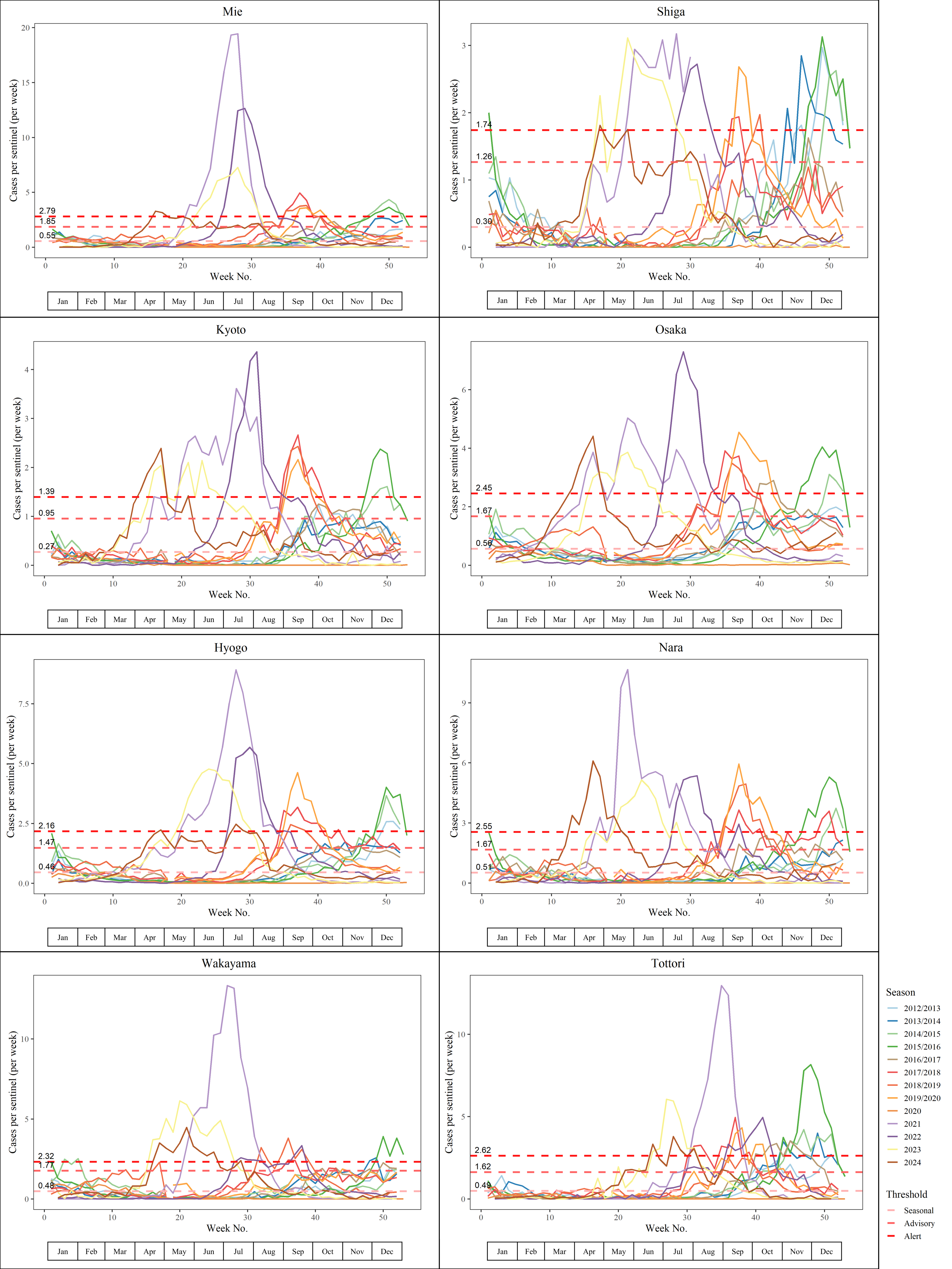

Supplement: Supplementary file 1 — Figure S1. RSV Epidemic curve with finalized thresholds nationwide and by prefecture in Japan, 2012–2024. [file PED-68-e70307-s004.zip › ped70307-sup-0004-FigureS1-S4@Supp_Figure1_4_highres.tif]

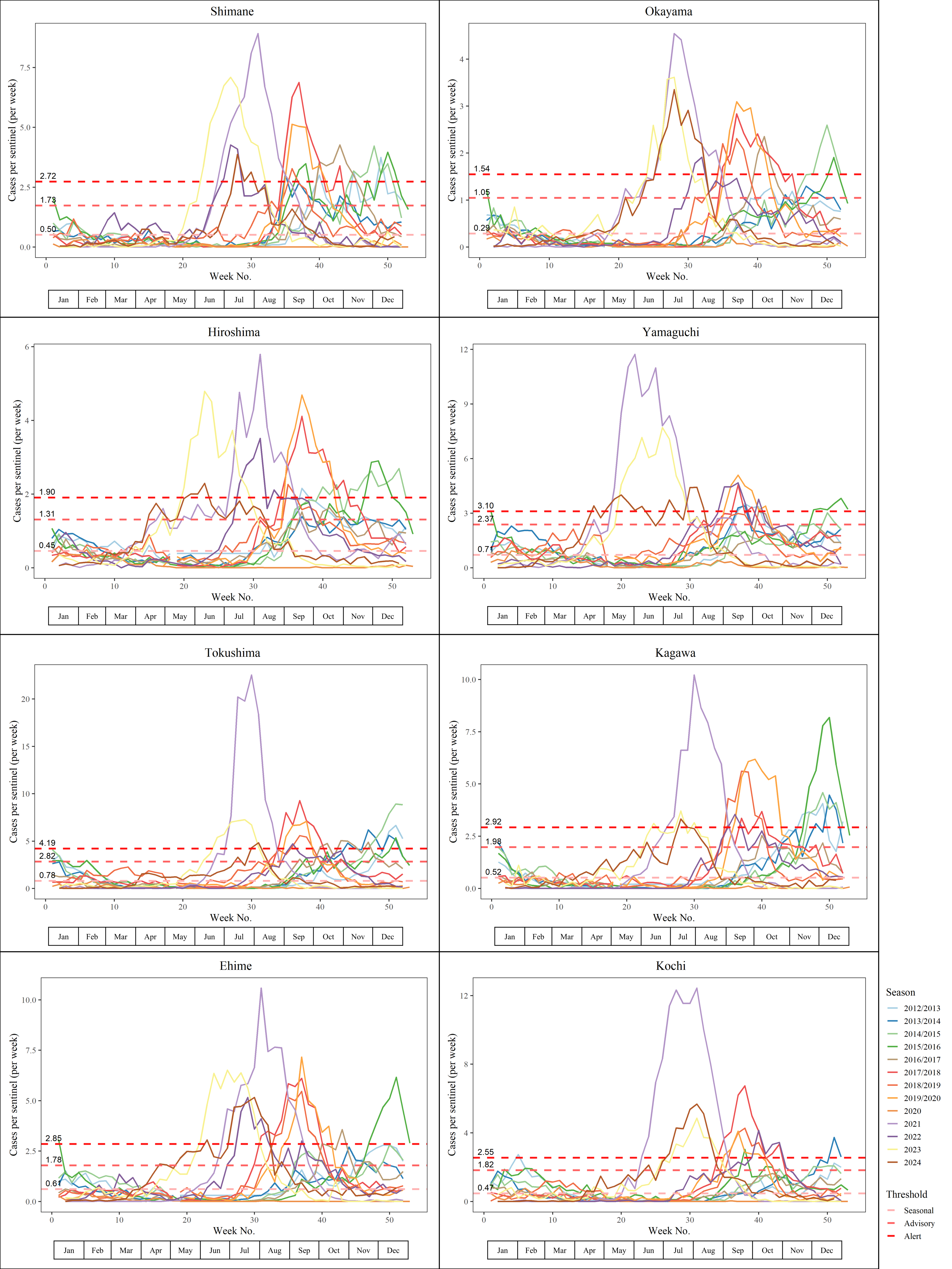

Supplement: Supplementary file 1 — Figure S1. RSV Epidemic curve with finalized thresholds nationwide and by prefecture in Japan, 2012–2024. [file PED-68-e70307-s004.zip › ped70307-sup-0005-FigureS1-S5@Supp_Figure1_5_highres.tif]

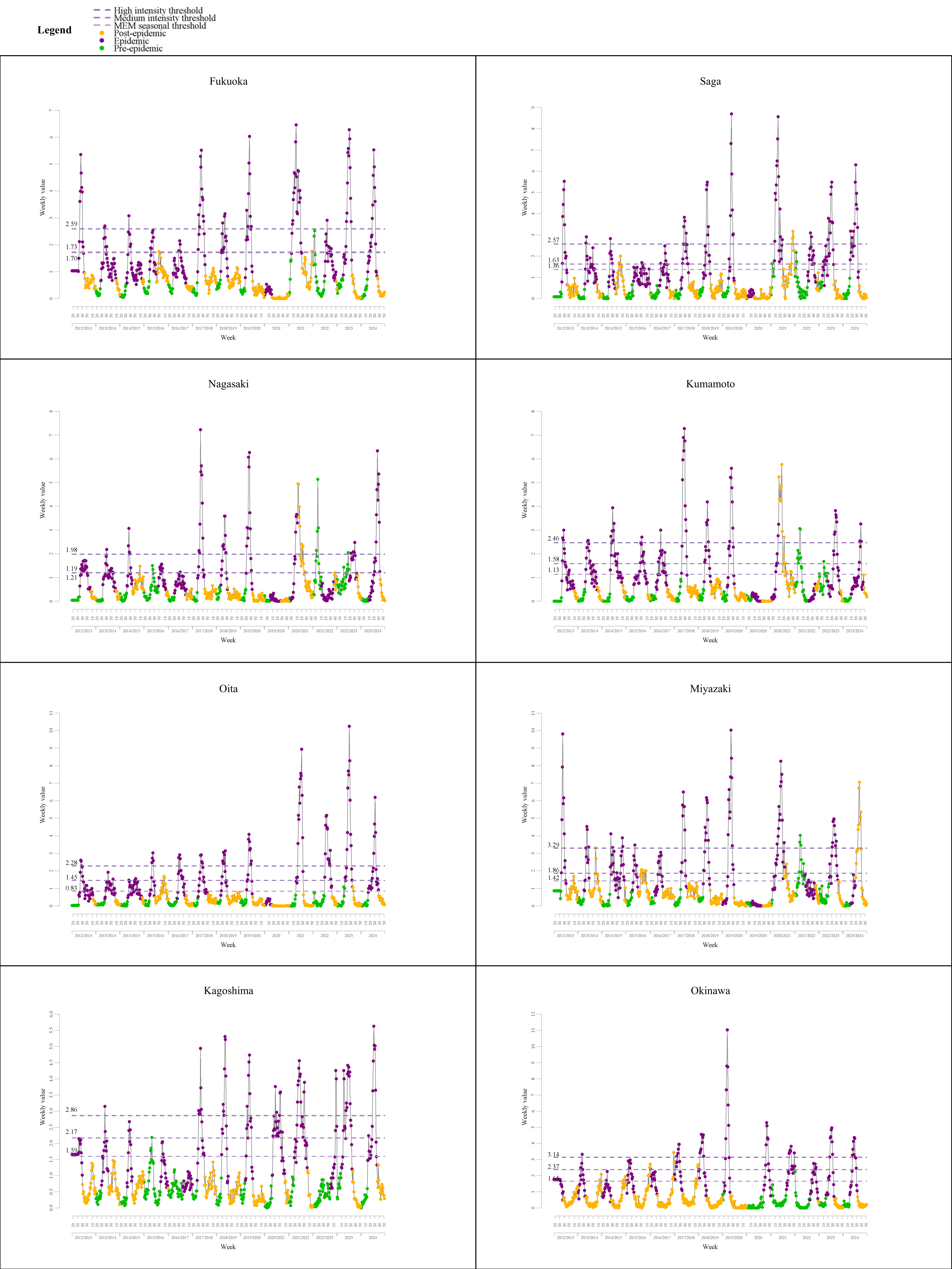

Supplement: Supplementary file 2 — Figure S2. MEM with their thresholds. [file PED-68-e70307-s005.zip › ped70307-sup-0012-FigureS2-S6@Supp_Figure2_6_highres.tif]

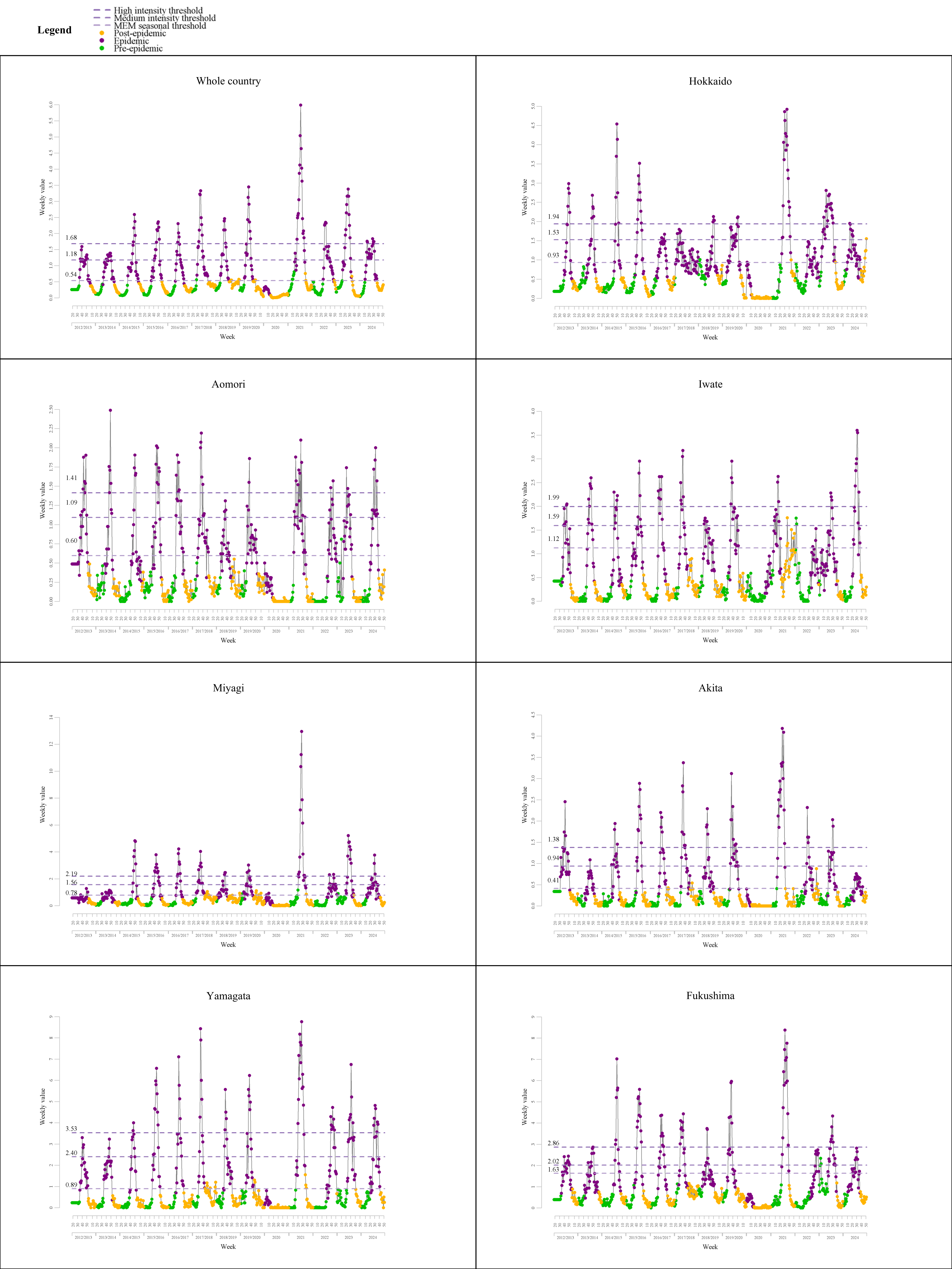

Supplement: Supplementary file 2 — Figure S2. MEM with their thresholds. [file PED-68-e70307-s005.zip › ped70307-sup-0007-FigureS2.tif]

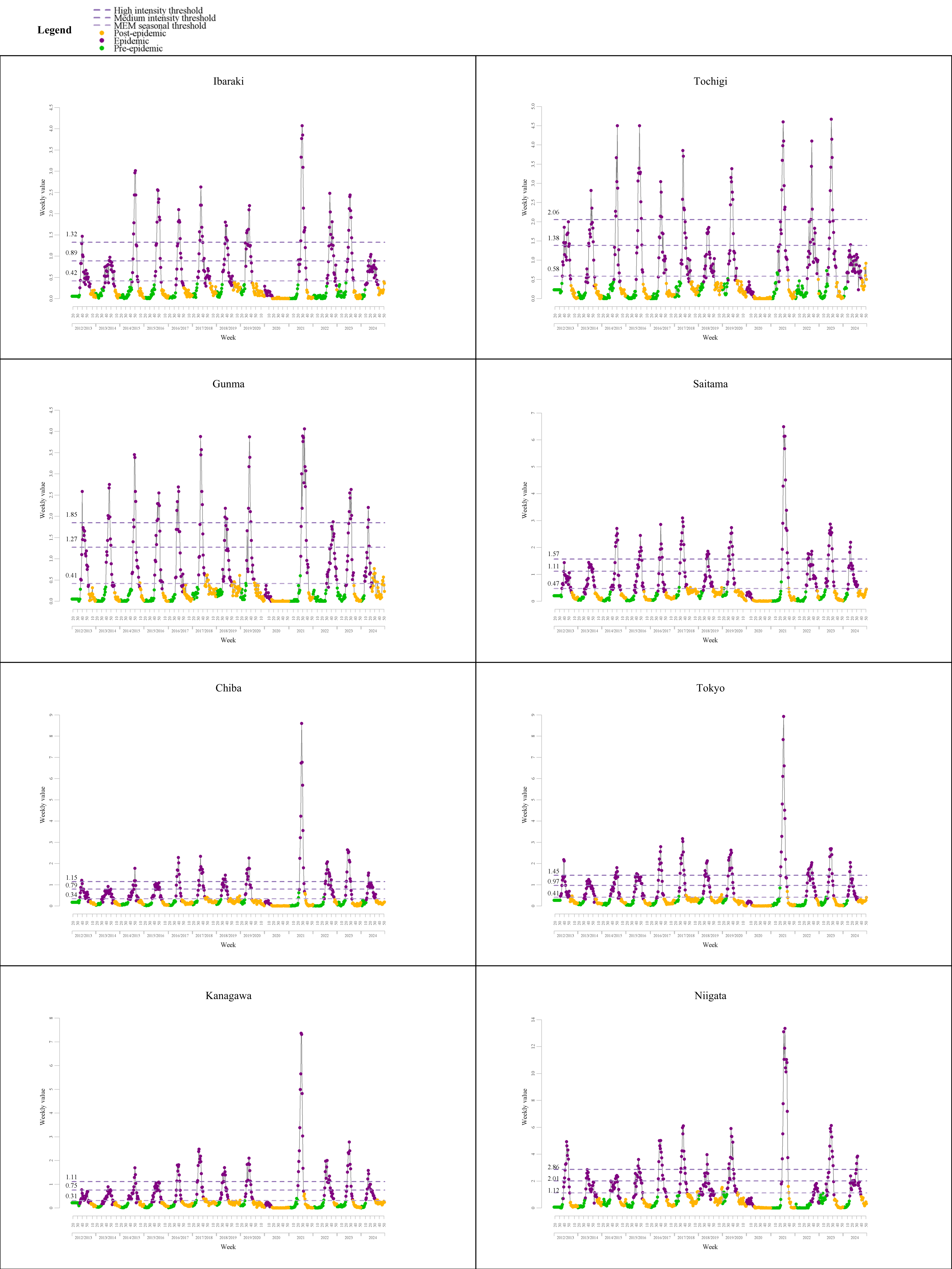

Supplement: Supplementary file 2 — Figure S2. MEM with their thresholds. [file PED-68-e70307-s005.zip › ped70307-sup-0008-FigureS2-S2@Supp_Figure2_2_highres.tif]

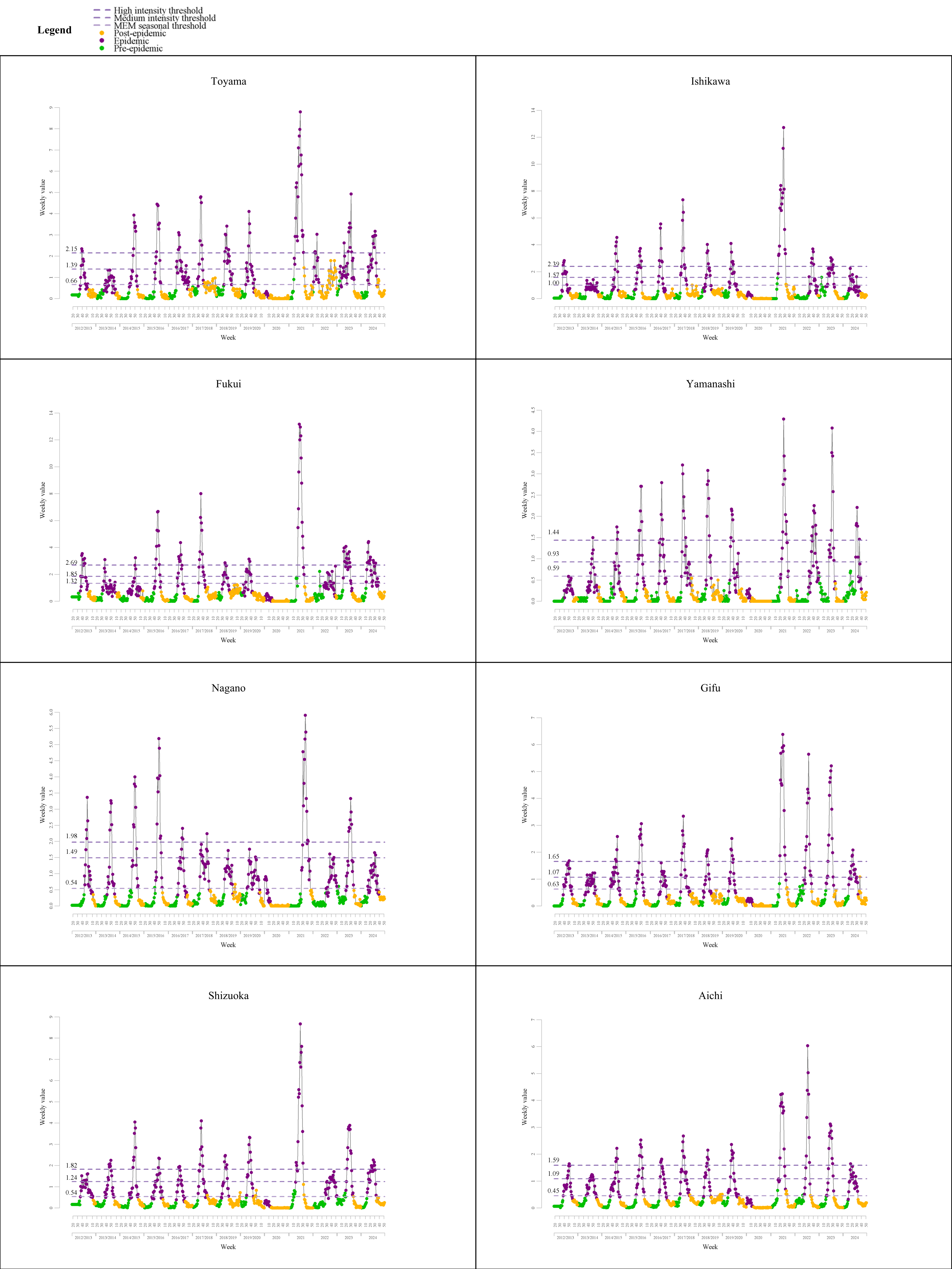

Supplement: Supplementary file 2 — Figure S2. MEM with their thresholds. [file PED-68-e70307-s005.zip › ped70307-sup-0009-FigureS2-S3@Supp_Figure2_3_highres.tif]

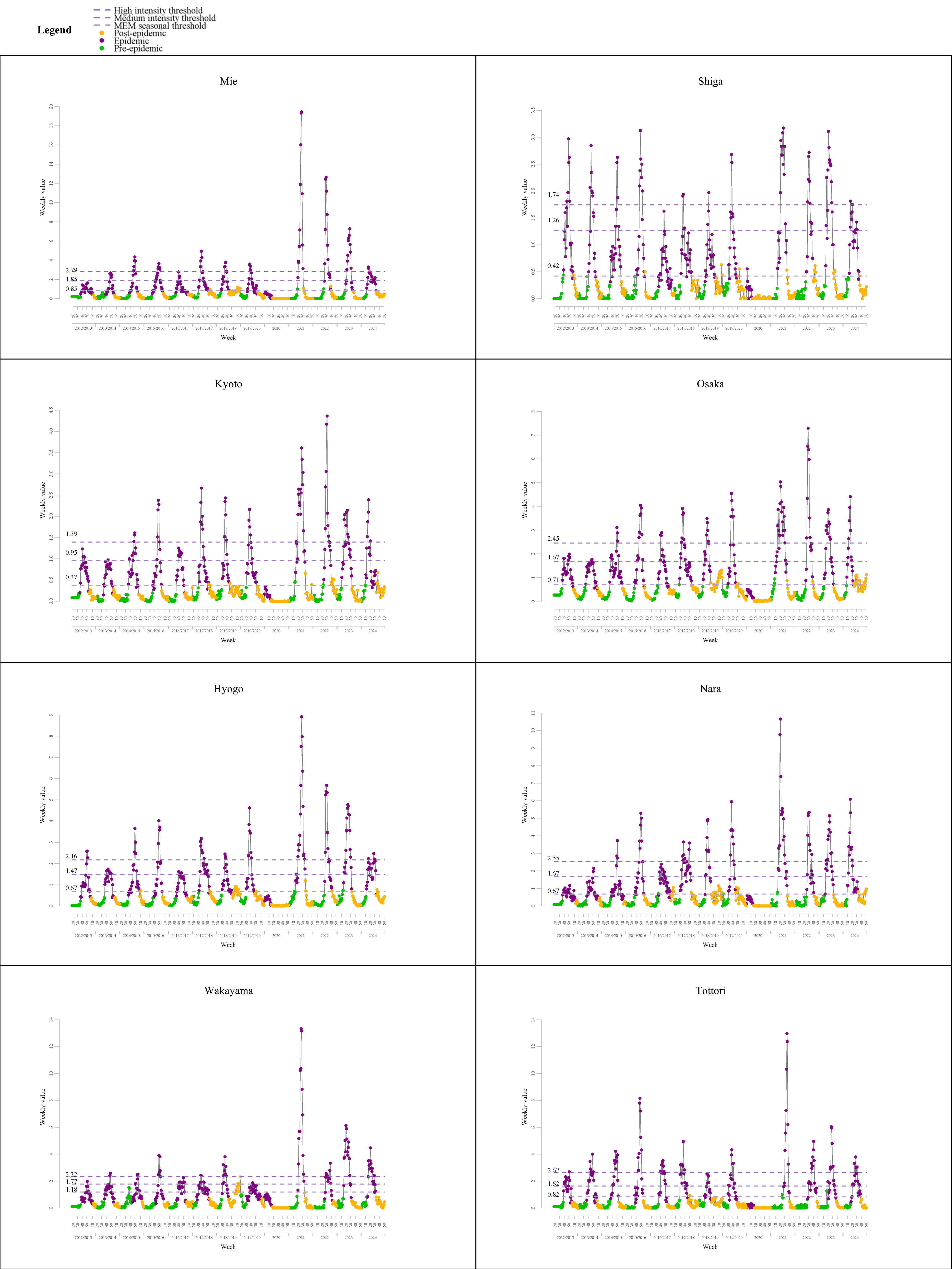

Supplement: Supplementary file 2 — Figure S2. MEM with their thresholds. [file PED-68-e70307-s005.zip › ped70307-sup-0010-FigureS2-S4@Supp_Figure2_4_highres.tif]

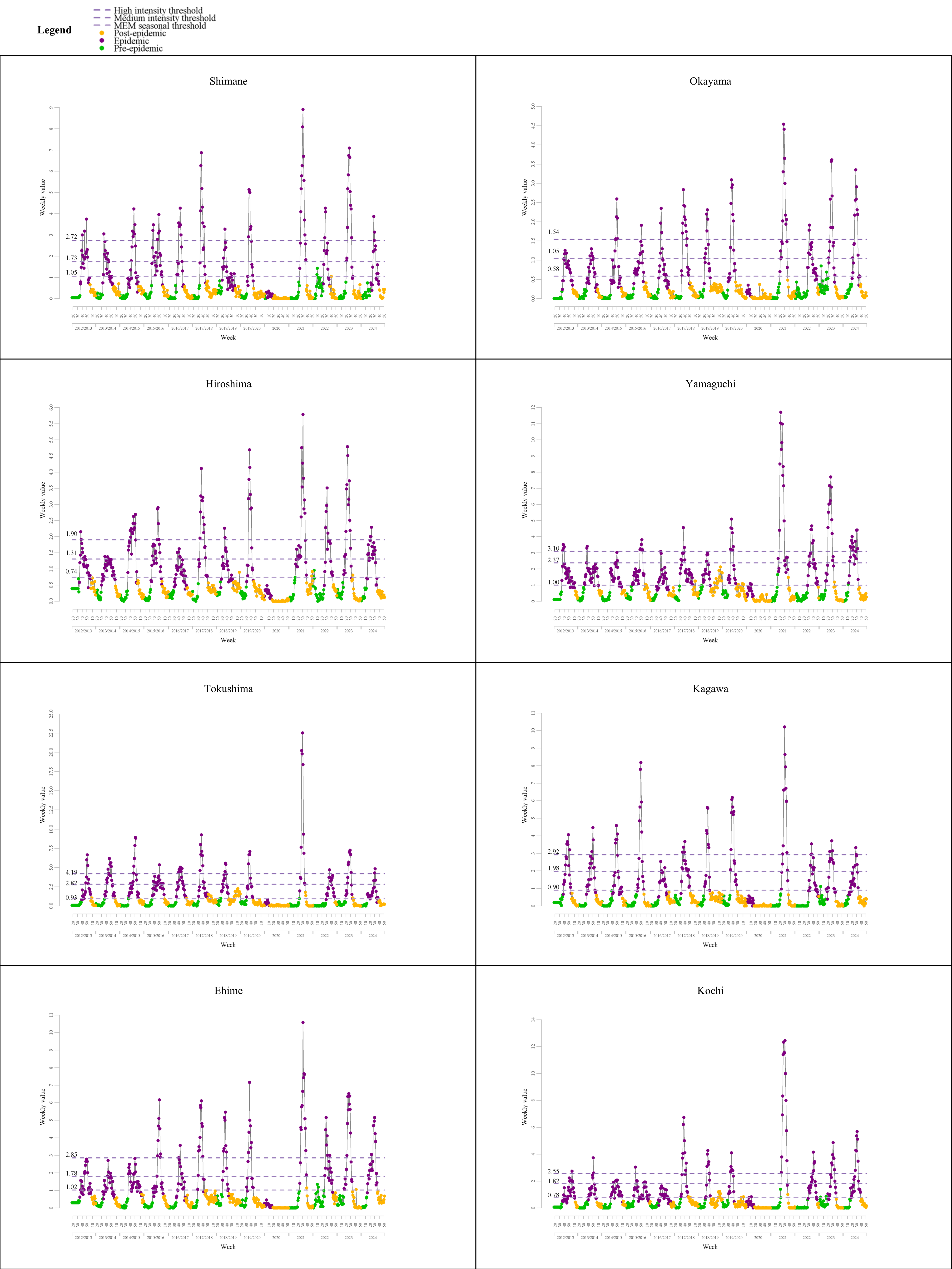

Supplement: Supplementary file 2 — Figure S2. MEM with their thresholds. [file PED-68-e70307-s005.zip › ped70307-sup-0011-FigureS2-S5@Supp_Figure2_5_highres.tif]

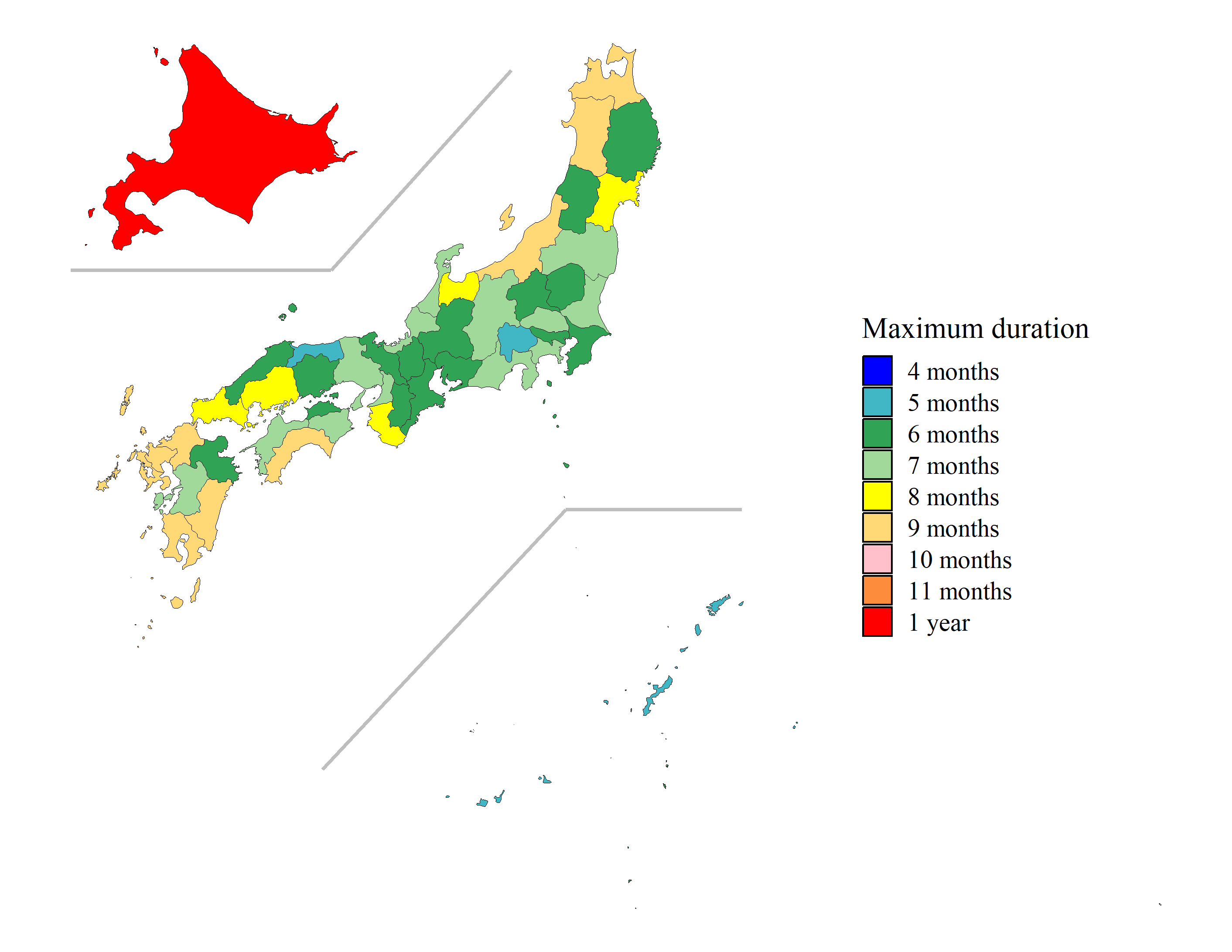

Supplement: Supplementary file 3 — Figure S3. Map of Japan showing the minimum and maximum lengths of the epidemic seasons. [file PED-68-e70307-s003.zip › ped70307-sup-0014-FigureS3@Supp_Figure3b_highres.tif]

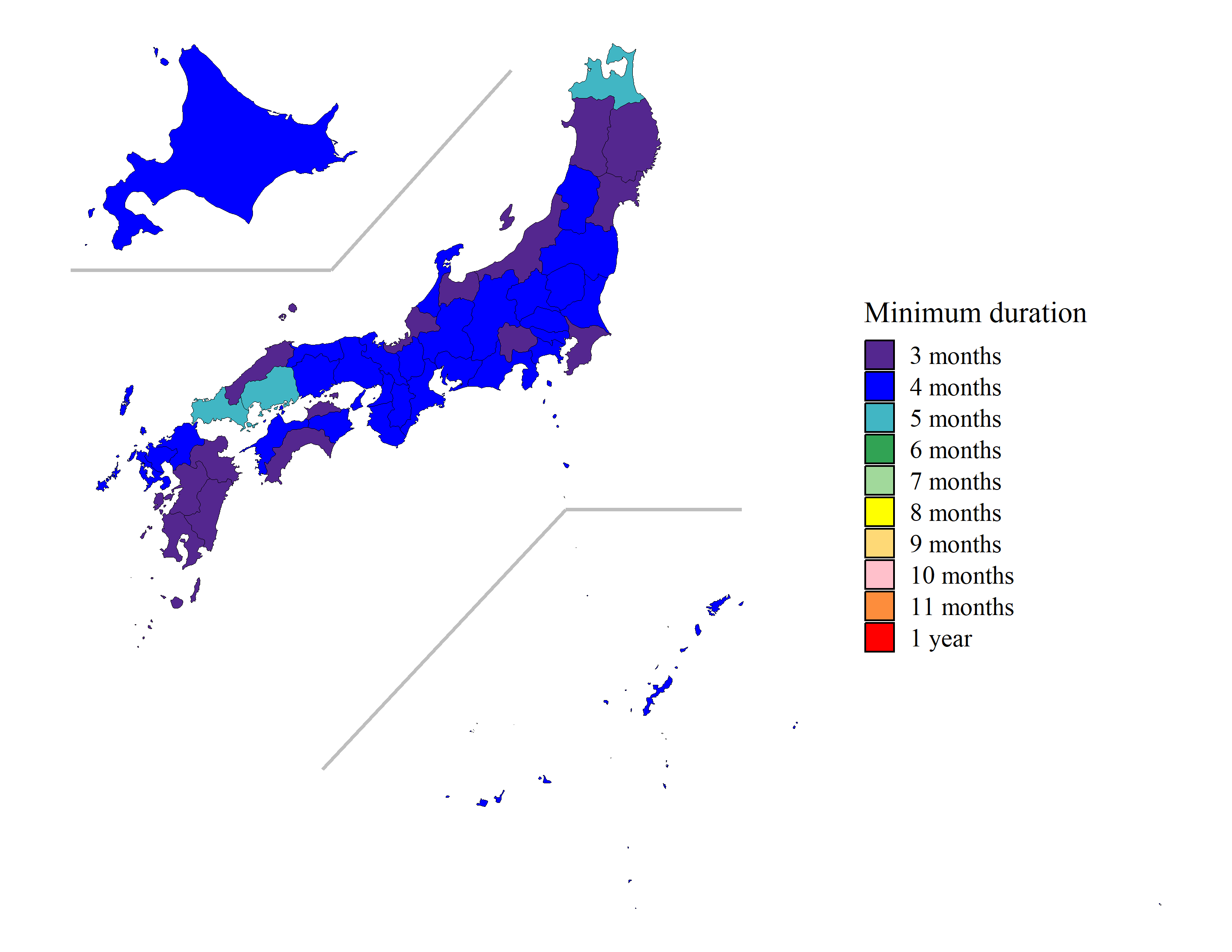

Supplement: Supplementary file 3 — Figure S3. Map of Japan showing the minimum and maximum lengths of the epidemic seasons. [file PED-68-e70307-s003.zip › ped70307-sup-0013-FigureS3.tif]

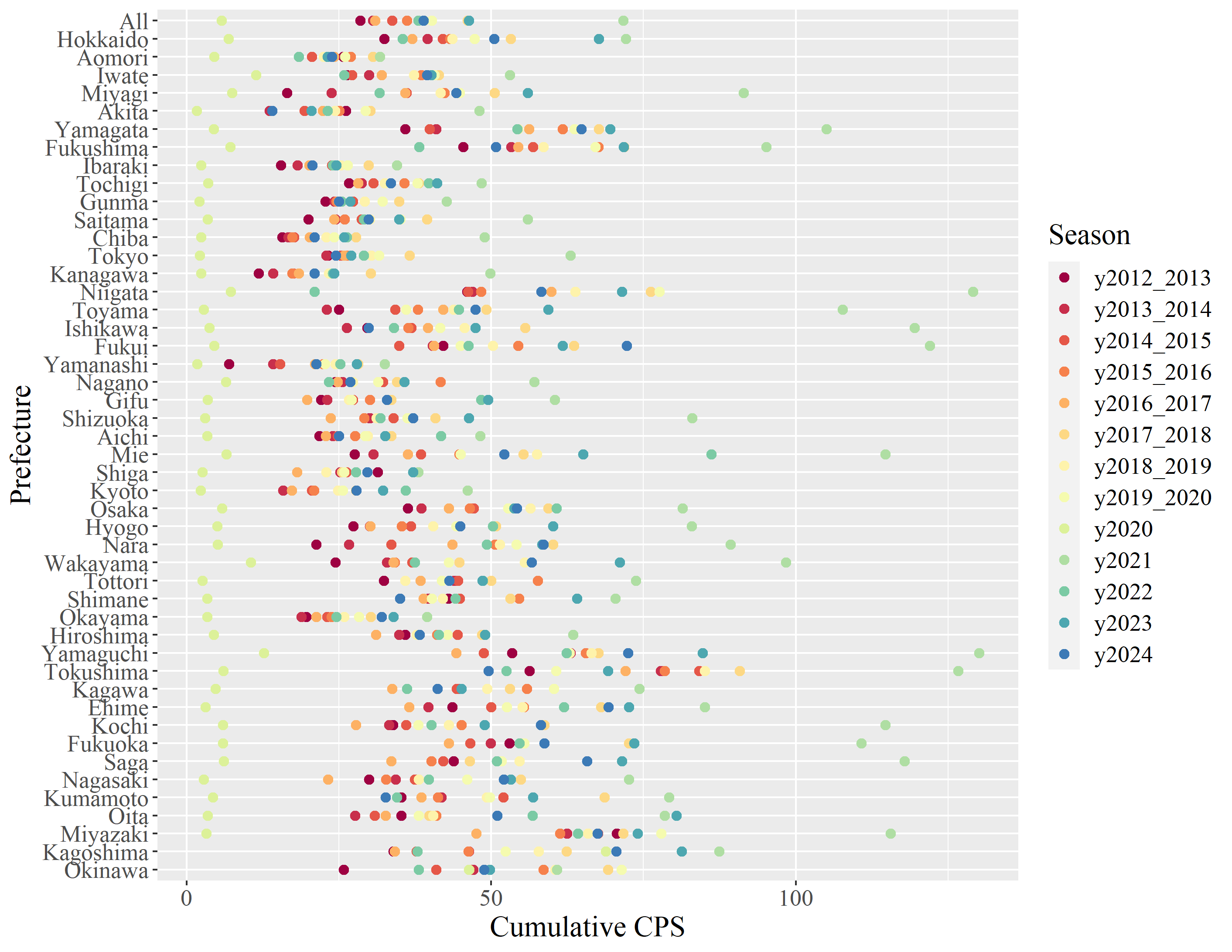

Supplement: Supplementary file 4 — Figure S4. Cumulative cases per sentinel. [file PED-68-e70307-s002.zip › ped70307-sup-0016-FigureS4@Supp_Figure4b_highres.tif]

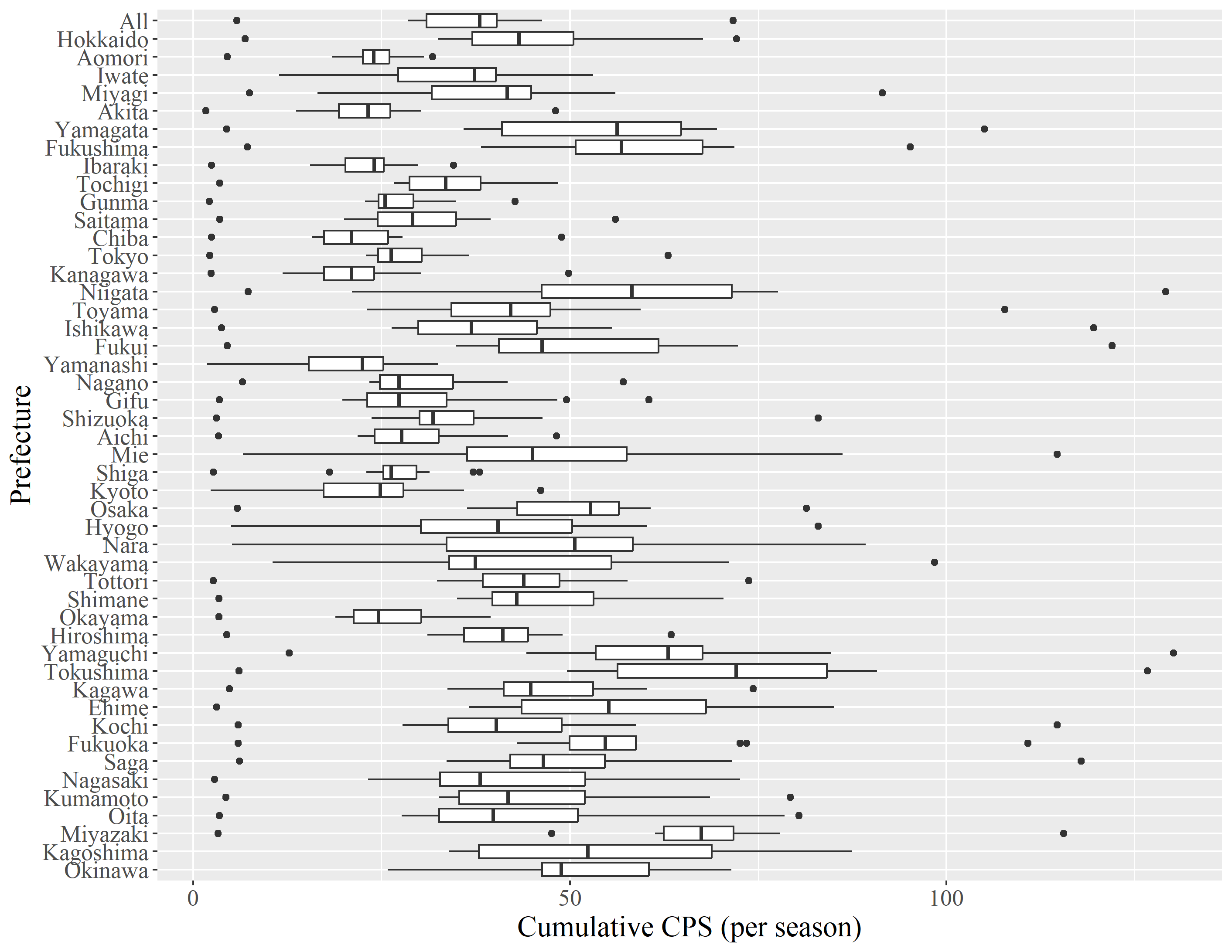

Supplement: Supplementary file 4 — Figure S4. Cumulative cases per sentinel. [file PED-68-e70307-s002.zip › ped70307-sup-0015-FigureS4.tif]
