# Supplementary material for: Spatial and temporal variability of respiratory syncytial virus disease seasonality in Japan, 2012–2024
Source: Pediatr Int. 2025 Dec 27;68(1):e70307. doi: 10.1111/ped.70307 (PMC12743260; doi:10.1111/ped.70307)
Supplement: Supplementary file 5 — Appendix S1. [file PED-68-e70307-s001.docx]

Supplementary Figure Legends and Footnotes

Supplementary Figure 1. RSV Epidemic curve with finalized thresholds nationwide and by prefecture in Japan, 2012 – 2024

Supplementary Figure 2. MEM with their thresholds

Supplementary Figure 3. Map of Japan showing the minimum and maximum lengths of the epidemic seasons

1. Minimum length of the epidemic seasons during 2012/2013 – 2024 seasons

Footnote: For Nagasaki, Kumamoto and Miyazaki, the time window was from week 45 to week 44 of the following year for the 2020/2021 - 2023/2024 seasons, while for Kagoshima, the time window was from week 18 to week 17 of the following year during the 2020/2021 - 2022/2023 seasons, then week 1 - 52 thereafter.

(b) Maximum length of the epidemic seasons during 2012/2013 – 2024 seasons

Footnote: For Nagasaki, Kumamoto and Miyazaki, the time window was from week 45 to week 44 of the following year for the 2020/2021 - 2023/2024 seasons, while for Kagoshima, the time window was from week 18 to week 17 of the following year during the 2020/2021 - 2022/2023 seasons, then week 1 - 52 thereafter.

**Supplementary Figure 4.** Cumulative cases per sentinel

1. Variability

Footnote: In the figure, the left and right sides of the box represent the 25th and 75th percentiles, respectively. The vertical line inside the box represents the median. Whiskers extend to the most extreme data points not considered outliers (i.e., greater than 1.5 times the interquartile range from the 25th and 75th percentiles, respectively), and the dots represent outliers. For Nagasaki, Kumamoto and Miyazaki, the time window was from week 45 to week 44 of the following year for the 2020/2021 - 2023/2024 seasons, while for Kagoshima, the time window was from week 18 to week 17 of the following year during the 2020/2021 - 2022/2023 seasons, then week 1 - 52 thereafter.

(b) Transitions across the study period

Footnote: For Nagasaki, Kumamoto and Miyazaki, the time window was from week 45 to week 44 of the following year for the 2020/2021 - 2023/2024 seasons, while for Kagoshima, the time window was from week 18 to week 17 of the following year during the 2020/2021 - 2022/2023 seasons, then week 1 - 52 thereafter.

Supplementary Table 1. Threshold values by method

| **National / prefecture** | **Seasonal - previous report: periodic oscillation** | **ROC method** | **1.2% fixed threshold method** | **MEM - epidemic threshold** | **MEM - medium intensity (20%)** | **MEM - high intensity (40%)** |
| --- | --- | --- | --- | --- | --- | --- |
| All |  | 0.330 | 0.418 | 0.538 | 1.178 | 1.683 |
| Hokkaido | 0.5 | 0.772 | 0.501 | 0.931 | 1.527 | 1.939 |
| *Tohoku* |  |  |  |  |  |  |
| Aomori | 0.49 | 0.252 | 0.277 | 0.596 | 1.092 | 1.413 |
| Iwate | 0.53 | 0.378 | 0.396 | 1.124 | 1.593 | 1.994 |
| Miyagi | 0.47 | 0.812 | 0.439 | 0.779 | 1.557 | 2.194 |
| Akita | 0.31 | 0.259 | 0.254 | 0.413 | 0.937 | 1.378 |
| Yamagata | 0.72 | 0.432 | 0.617 | 0.894 | 2.399 | 3.531 |
| Fukushima | 0.76 | 0.937 | 0.648 | 1.631 | 2.016 | 2.864 |
| *Kanto* |  |  |  |  |  |  |
| Ibaraki | 0.33 | 0.205 | 0.256 | 0.417 | 0.886 | 1.324 |
| Tochigi | 0.46 | 0.270 | 0.379 | 0.580 | 1.381 | 2.055 |
| Gumma | 0.37 | 0.222 | 0.300 | 0.415 | 1.271 | 1.848 |
| Saitama | 0.35 | 0.331 | 0.328 | 0.472 | 1.113 | 1.569 |
| Chiba | 0.26 | 0.281 | 0.240 | 0.341 | 0.792 | 1.147 |
| Tokyo | 0.31 | 0.304 | 0.308 | 0.410 | 0.966 | 1.447 |
| Kanagawa | 0.22 | 0.242 | 0.231 | 0.310 | 0.754 | 1.110 |
| *Chubu* |  |  |  |  |  |  |
| Niigata | 0.74 | 1.188 | 0.624 | 1.121 | 2.008 | 2.863 |
| Toyama | 0.61 | 0.465 | 0.448 | 0.659 | 1.391 | 2.151 |
| Ishikawa | 0.59 | 0.412 | 0.427 | 0.996 | 1.574 | 2.389 |
| Fukui | 0.68 | 0.665 | 0.560 | 1.323 | 1.850 | 2.693 |
| Yamanashi | 0.25 | 0.378 | 0.232 | 0.587 | 0.926 | 1.437 |
| Nagano | 0.4 | 0.195 | 0.335 | 0.540 | 1.490 | 1.979 |
| Gifu | 0.31 | 0.282 | 0.344 | 0.627 | 1.067 | 1.654 |
| Shizuoka | 0.39 | 0.365 | 0.376 | 0.537 | 1.243 | 1.818 |
| Aichi | 0.4 | 0.202 | 0.317 | 0.450 | 1.085 | 1.589 |
| Mie | 0.64 | 0.369 | 0.548 | 0.845 | 1.848 | 2.788 |
| *Kinki* |  |  |  |  |  |  |
| Shiga | 0.45 | 0.352 | 0.300 | 0.417 | 1.265 | 1.740 |
| Kyoto | 0.23 | 0.169 | 0.270 | 0.374 | 0.953 | 1.393 |
| Osaka | 0.61 | 0.495 | 0.558 | 0.710 | 1.668 | 2.453 |
| Hyogo | 0.45 | 0.332 | 0.456 | 0.665 | 1.474 | 2.165 |
| Nara | 0.56 | 0.351 | 0.514 | 0.675 | 1.668 | 2.546 |
| Wakayama | 0.48 | 0.475 | 0.483 | 1.182 | 1.766 | 2.318 |
| *Chugoku* |  |  |  |  |  |  |
| Tottori | 0.58 | 0.656 | 0.489 | 0.820 | 1.625 | 2.619 |
| Shimane | 0.65 | 0.457 | 0.504 | 1.046 | 1.732 | 2.725 |
| Okayama | 0.28 | 0.240 | 0.285 | 0.579 | 1.045 | 1.544 |
| Hiroshima | 0.46 | 0.298 | 0.455 | 0.736 | 1.306 | 1.902 |
| Yamaguchi | 0.6 | 0.373 | 0.706 | 0.996 | 2.371 | 3.097 |
| *Shikoku* |  |  |  |  |  |  |
| Tokushima | 1 | 0.500 | 0.784 | 0.932 | 2.824 | 4.195 |
| Kagawa | 0.61 | 0.359 | 0.516 | 0.897 | 1.976 | 2.918 |
| Ehime | 0.54 | 0.478 | 0.612 | 1.016 | 1.784 | 2.851 |
| Kochi | 0.43 | 0.490 | 0.470 | 0.785 | 1.816 | 2.547 |
| *Kyushu* |  |  |  |  |  |  |
| Fukuoka | 0.53 | 0.604 | 0.636 | 1.704 | 1.731 | 2.587 |
| Saga | 0.7 | 0.466 | 0.550 | 1.362 | 1.627 | 2.568 |
| Nagasaki^*^ | 0.39 | 0.345 | 0.478 | 1.208 | 1.193 | 1.978 |
| Kumamoto^*^ | 0.53 | 0.450 | 0.550 | 1.130 | 1.583 | 2.463 |
| Oita | 0.47 | 0.308 | 0.478 | 0.833 | 1.450 | 2.276 |
| Miyazaki^*^ | 0.81 | 0.395 | 0.796 | 1.421 | 1.861 | 3.293 |
| Kagoshima^**^ | 0.38 | 0.605 | 0.651 | 1.594 | 2.168 | 2.858 |
| Okinawa | 0.5 | 0.470 | 0.626 | 1.651 | 2.372 | 3.136 |

*Time window is from week 18 to week 17 of the following year by the 2019/2020 season, then from week 45 to week 44 of the following year thereafter.

**Time window is from week 18 to week 17 of the following year by the 2022/2023 season, then from week 1 to 52 thereafter.

Supplementary Table 2. Length of seasons nationally and by prefecture

| National / prefecture | Mean (SD) | Median (Q1, Q3) | 2012 / 2013 | 2013 / 2014 | 2014 / 2015 | 2015 / 2016 | 2016 / 2017 | 2017 / 2018 | 2018 / 2019 | 2019 / 2020 | 2020 | 2021 | 2022 | 2023 | 2024 |
| --- | --- | --- | --- | --- | --- | --- | --- | --- | --- | --- | --- | --- | --- | --- | --- |
| All | 26.1 (3.3) | 25.5 (24.0, 27.2) | 24 | 24 | 27 | 24 | 20 | 30 | 26 | 25 | NA | 33 | 27 | 25 | 28 |
| Hokkaido | 27.0 (10.9) | 28.0 (22.0, 33.0) | 17 | 22 | 16 | 23 | 24 | 51 | 29 | 33 | 7 | 32 | 28 | 34 | 35 |
| *Tohoku* |  |  |  |  |  |  |  |  |  |  |  |  |  |  |  |
| Aomori | 21.8 (7.7) | 22.0 (19.0, 24.0) | 37 | 18 | 22 | 20 | 19 | 28 | 24 | 24 | 3 | 28 | 19 | 22 | 20 |
| Iwate | 19.4 (6.0) | 20.0 (18.0, 24.0) | 19 | 18 | 13 | 26 | 18 | 20 | 25 | 24 | 5 | 24 | 14 | 25 | 21 |
| Miyagi | 21.5 (8.6) | 23.0 (16.0, 24.0) | 15 | 18 | 13 | 24 | 16 | 33 | 24 | 33 | 3 | 24 | 22 | 23 | 32 |
| Akita | 19.2 (7.9) | 19.0 (17.0, 22.0) | 37 | 18 | 13 | 17 | 17 | 23 | 24 | 22 | 1 | 20 | 20 | 18 | 19 |
| Yamagata | 18.0 (5.7) | 19.0 (17.0, 22.0) | 18 | 18 | 15 | 17 | 19 | 23 | 19 | 17 | 1 | 24 | 19 | 22 | 22 |
| Fukushima | 23.0 (4.8) | 22.0 (20.0, 28.0) | 23 | 29 | 17 | 20 | 20 | 29 | 28 | 21 | NA | 21 | 15 | 25 | 28 |
| *Kanto* |  |  |  |  |  |  |  |  |  |  |  |  |  |  |  |
| Ibaraki | 19.2 (7.3) | 19.0 (17.0, 24.0) | 22 | 24 | 14 | 14 | 17 | 29 | 24 | 18 | 1 | 19 | 22 | 17 | 28 |
| Tochigi | 19.5 (6.8) | 20.0 (18.0, 25.0) | 20 | 18 | 15 | 15 | 18 | 24 | 25 | 25 | 1 | 25 | 25 | 18 | 25 |
| Gunma | 17.5 (5.6) | 18.0 (16.0, 21.0) | 18 | 19 | 18 | 16 | 15 | 22 | 22 | 21 | 1 | 18 | 23 | 19 | 16 |
| Saitama | 20.3 (6.6) | 21.0 (19.0, 23.0) | 21 | 20 | 23 | 18 | 17 | 21 | 27 | 26 | 1 | 19 | 26 | 23 | 22 |
| Chiba | 17.8 (6.1) | 19.0 (17.0, 22.0) | 19 | 19 | 22 | 23 | 12 | 24 | 18 | 17 | 1 | 17 | 23 | 16 | 21 |
| Tokyo | 20.7 (2.2) | 20.5 (19.0, 22.2) | 21 | 22 | 23 | 23 | 17 | 20 | 22 | 18 | NA | 19 | 24 | 20 | 19 |
| Kanagawa | 18.3 (5.9) | 18.0 (18.0, 21.0) | 19 | 18 | 17 | 21 | 18 | 27 | 20 | 18 | 1 | 17 | 22 | 18 | 22 |
| *Chubu* |  |  |  |  |  |  |  |  |  |  |  |  |  |  |  |
| Niigata | 20.5 (8.3) | 21.0 (19.0, 26.0) | 16 | 19 | 22 | 21 | 20 | 26 | 35 | 29 | 1 | 19 | 12 | 21 | 26 |
| Toyama | 20.7 (6.3) | 18.5 (18.0, 25.0) | 18 | 11 | 18 | 14 | 28 | 18 | 20 | 18 | NA | 25 | 19 | 34 | 25 |
| Ishikawa | 18.4 (6.6) | 18.0 (16.0, 23.0) | 18 | 27 | 16 | 16 | 17 | 15 | 25 | 20 | 1 | 25 | 16 | 23 | 20 |
| Fukui | 17.3 (6.8) | 18.0 (16.0, 21.0) | 17 | 13 | 10 | 16 | 16 | 21 | 18 | 18 | 1 | 21 | 23 | 22 | 29 |
| Yamanashi | 14.7 (5.0) | 15.0 (13.0, 18.0) | 12 | 12 | 13 | 19 | 17 | 21 | 15 | 13 | 1 | 18 | 19 | 16 | 15 |
| Nagano | 19.6 (5.5) | 19.0 (17.0, 23.0) | 19 | 17 | 15 | 18 | 17 | 25 | 20 | 30 | 8 | 23 | 19 | 18 | 26 |
| Gifu | 20.2 (3.0) | 19.5 (18.0, 22.0) | 20 | 26 | 22 | 18 | 17 | 24 | 18 | 16 | NA | 19 | 22 | 19 | 22 |
| Shizuoka | 21.9 (3.9) | 21.0 (20.0, 25.2) | 28 | 21 | 21 | 25 | 16 | 24 | 17 | 17 | NA | 26 | 26 | 21 | 21 |
| Aichi | 19.2 (6.0) | 21.0 (18.0, 22.0) | 21 | 23 | 22 | 19 | 17 | 25 | 19 | 24 | 1 | 22 | 17 | 18 | 21 |
| Mie | 18.8 (5.8) | 21.0 (17.0, 22.0) | 23 | 15 | 18 | 24 | 24 | 22 | 17 | 21 | 2 | 16 | 19 | 21 | 22 |
| *Kinki* |  |  |  |  |  |  |  |  |  |  |  |  |  |  |  |
| Shiga | 19.8 (6.2) | 21.0 (18.0, 23.0) | 26 | 17 | 23 | 19 | 20 | 23 | 23 | 18 | 2 | 16 | 23 | 21 | 26 |
| Kyoto | 19.7 (6.2) | 22.0 (17.0, 24.0) | 17 | 18 | 22 | 19 | 17 | 25 | 17 | 23 | 2 | 24 | 22 | 26 | 24 |
| Osaka | 23.5 (4.3) | 23.5 (21.0, 25.5) | 24 | 18 | 27 | 22 | 23 | 30 | 18 | 25 | NA | 30 | 17 | 23 | 25 |
| Hyogo | 21.4 (7.4) | 23.0 (18.0, 27.0) | 18 | 16 | 28 | 18 | 23 | 29 | 25 | 23 | 1 | 27 | 19 | 24 | 27 |
| Nara | 19.1 (6.2) | 20.0 (18.0, 23.0) | 18 | 20 | 23 | 21 | 23 | 25 | 16 | 16 | 1 | 23 | 19 | 19 | 24 |
| Wakayama | 19.7 (6.1) | 18.0 (16.0, 20.0) | 14 | 20 | 20 | 16 | 18 | 29 | 17 | 34 | 12 | 15 | 18 | 20 | 23 |
| *Chugoku* |  |  |  |  |  |  |  |  |  |  |  |  |  |  |  |
| Tottori | 17.4 (2.4) | 18.0 (15.8, 19.0) | 14 | 18 | 18 | 15 | 16 | 19 | 14 | 19 | NA | 16 | 21 | 19 | 20 |
| Shimane | 17.4 (4.1) | 16.0 (14.8, 20.5) | 22 | 22 | 20 | 26 | 16 | 14 | 12 | 14 | NA | 16 | 16 | 16 | 15 |
| Okayama | 18.4 (5.9) | 19.0 (18.0, 22.0) | 22 | 19 | 23 | 23 | 19 | 18 | 14 | 17 | 1 | 20 | 21 | 18 | 24 |
| Hiroshima | 21.5 (7.2) | 23.0 (19.0, 25.0) | 19 | 31 | 24 | 26 | 23 | 21 | 25 | 18 | 1 | 28 | 19 | 21 | 23 |
| Yamaguchi | 24.2 (7.5) | 25.0 (21.0, 29.0) | 29 | 33 | 25 | 33 | 23 | 30 | 21 | 19 | 4 | 27 | 24 | 21 | 25 |
| *Shikoku* |  |  |  |  |  |  |  |  |  |  |  |  |  |  |  |
| Tokushima | 18.6 (6.5) | 20.0 (16.0, 23.0) | 20 | 24 | 23 | 27 | 23 | 22 | 17 | 14 | 1 | 15 | 19 | 16 | 21 |
| Kagawa | 17.5 (5.8) | 18.0 (17.0, 21.0) | 19 | 21 | 21 | 18 | 18 | 23 | 13 | 15 | 1 | 18 | 23 | 17 | 21 |
| Ehime | 21.6 (4.2) | 21.0 (18.8, 23.5) | 26 | 23 | 30 | 23 | 17 | 21 | 18 | 15 | NA | 19 | 21 | 21 | 25 |
| Kochi | 19.3 (7.9) | 19.0 (17.0, 23.0) | 17 | 19 | 25 | 35 | 19 | 23 | 12 | 13 | 3 | 17 | 21 | 19 | 28 |
| *Kyushu* |  |  |  |  |  |  |  |  |  |  |  |  |  |  |  |
| Fukuoka | 25.1 (6.2) | 24.5 (19.0, 27.5) | 29 | 35 | 19 | 24 | 27 | 19 | 19 | 17 | NA | 36 | 27 | 25 | 24 |
| Saga | 20.7 (7.2) | 17.5 (15.0, 26.2) | 14 | 26 | 15 | 36 | 16 | 15 | 16 | 14 | NA | 27 | 19 | 29 | 21 |
| Nagasaki* | 21.2 (6.9) | 19.0 (16.0, 23.8) | 23 | 26 | 20 | 16 | 21 | 16 | 16 | 15 | NA | 29 | 16 | 38 | 18 |
| Kumamoto* | 20.1 (5.9) | 18.0 (16.0, 25.2) | 12 | 25 | 28 | 30 | 21 | 17 | 16 | 17 | NA | 26 | 16 | 14 | 19 |
| Oita | 18.7 (4.4) | 18.5 (15.5, 22.0) | 13 | 22 | 26 | 13 | 16 | 21 | 16 | 14 | NA | 18 | 19 | 22 | 24 |
| Miyazaki* | 18.3 (6.2) | 16.5 (15.0, 19.5) | 14 | 15 | 17 | 17 | 13 | 15 | 16 | 15 | NA | 36 | 22 | 21 | 19 |
| Kagoshima** | 19.4 (8.2) | 18.0 (13.0, 24.0) | 24 | 15 | 12 | 13 | 9 | 19 | 16 | 18 | 30 | 35 | 10 | 29 | 22 |
| Okinawa | 17.5 (1.5) | 18.0 (16.0, 18.0) | 19 | 15 | 18 | 19 | 16 | 18 | 18 | 16 | 16 | 20 | 16 | 18 | 18 |

*Time window is from week 18 to week 17 of the following year by the 2019/2020 season, then from week 45 to week 44 of the following year thereafter.

**Time window is from week 18 to week 17 of the following year by the 2022/2023 season, then from week 1 to 52 thereafter

NA: Not applicable

Supplementary Table 3. Season onset and end by year and nationally/by prefecture

| **National/prefecture** | **2012/2013** | **2013/2014** | **2014/2015** | **2015/2016** | **2016/2017** | **2017/2018** | **2018/2019** | **2019/2020** | **2020** | **2021** | **2022** | **2023** | **2024** |
| --- | --- | --- | --- | --- | --- | --- | --- | --- | --- | --- | --- | --- | --- |
| All | (35, 6) | (35, 6) | (36, 10) | (35, 5) | (34, 1) | (28, 5) | (27, 52) | (28, 52) | (NA, NA) | (9, 41) | (24, 50) | (12, 36) | (11, 38) |
| *Tohoku* |  |  |  |  |  |  |  |  |  |  |  |  |  |
| Hokkaido | (40, 4) | (36, 5) | (43, 6) | (36, 5) | (31, 2) | (19, 17) | (33, 9) | (27, 7) | (1, 7) | (20, 51) | (25, 52) | (4, 37) | (4, 38) |
| Aomori | (18, 2) | (37, 2) | (42, 11) | (36, 2) | (33, 51) | (27, 2) | (26, 49) | (31, 2) | (13, 15) | (11, 38) | (34, 52) | (14, 35) | (20, 39) |
| Iwate | (35, 1) | (37, 2) | (42, 2) | (29, 1) | (34, 51) | (27, 46) | (28, 52) | (29, 52) | (49, 53) | (29, 52) | (37, 50) | (13, 37) | (21, 41) |
| Miyagi | (18, 32) | (35, 52) | (45, 5) | (34, 4) | (34, 49) | (25, 5) | (19, 42) | (20, 52) | (4, 6) | (15, 38) | (31, 52) | (15, 37) | (12, 43) |
| Akita | (18, 2) | (36, 1) | (42, 2) | (38, 1) | (36, 52) | (31, 1) | (28, 51) | (32, 1) | (1, 1) | (13, 32) | (21, 40) | (21, 38) | (25, 43) |
| Yamagata | (35, 52) | (35, 52) | (39, 1) | (36, 52) | (33, 51) | (31, 1) | (33, 51) | (29, 45) | (5, 5) | (15, 38) | (34, 52) | (21, 42) | (20, 41) |
| Fukushima | (34, 4) | (35, 11) | (43, 7) | (35, 1) | (34, 1) | (22, 50) | (25, 52) | (26, 46) | (NA, NA) | (21, 41) | (38, 52) | (15, 39) | (11, 38) |
| *Kanto* |  |  |  |  |  |  |  |  |  |  |  |  |  |
| Ibaraki | (36, 5) | (35, 6) | (44, 5) | (42, 2) | (34, 50) | (28, 4) | (30, 1) | (28, 45) | (1, 1) | (20, 38) | (30, 51) | (20, 36) | (10, 37) |
| Tochigi | (35, 2) | (35, 52) | (43, 5) | (40, 1) | (35, 52) | (30, 1) | (28, 52) | (28, 52) | (6, 6) | (14, 38) | (27, 51) | (20, 37) | (9, 33) |
| Gunma | (37, 2) | (39, 5) | (44, 9) | (41, 3) | (33, 47) | (31, 52) | (31, 52) | (29, 49) | (4, 4) | (25, 42) | (30, 52) | (19, 37) | (8, 23) |
| Saitama | (35, 3) | (35, 2) | (36, 6) | (40, 4) | (34, 50) | (27, 47) | (26, 52) | (27, 52) | (4, 4) | (22, 40) | (24, 49) | (13, 35) | (11, 32) |
| Chiba | (34, 52) | (34, 52) | (36, 5) | (35, 4) | (34, 45) | (29, 52) | (26, 43) | (27, 43) | (9, 9) | (21, 37) | (24, 46) | (18, 33) | (12, 32) |
| Tokyo | (32, 52) | (31, 52) | (36, 6) | (35, 4) | (31, 47) | (27, 46) | (25, 46) | (26, 43) | (NA, NA) | (19, 37) | (25, 48) | (15, 34) | (11, 29) |
| Kanagawa | (36, 2) | (35, 52) | (36, 52) | (35, 2) | (31, 48) | (26, 52) | (26, 45) | (27, 44) | (6, 6) | (19, 35) | (24, 45) | (19, 36) | (11, 32) |
| *Chubu* |  |  |  |  |  |  |  |  |  |  |  |  |  |
| Niigata | (37, 52) | (34, 52) | (36, 5) | (34, 1) | (33, 52) | (27, 52) | (18, 52) | (24, 52) | (6, 6) | (22, 40) | (39, 50) | (13, 33) | (15, 40) |
| Toyama | (35, 52) | (42, 52) | (38, 3) | (41, 1) | (35, 10) | (29, 46) | (33, 52) | (29, 46) | (NA, NA) | (11, 35) | (34, 52) | (4, 37) | (15, 39) |
| Ishikawa | (35, 52) | (35, 9) | (39, 2) | (37, 52) | (34, 50) | (31, 45) | (26, 50) | (31, 50) | (5, 5) | (13, 37) | (31, 46) | (14, 36) | (13, 32) |
| Fukui | (36, 52) | (32, 44) | (43, 52) | (40, 2) | (37, 52) | (31, 51) | (30, 47) | (28, 45) | (6, 6) | (16, 36) | (24, 46) | (12, 33) | (10, 38) |
| Yamanashi | (46, 5) | (41, 52) | (46, 6) | (41, 6) | (36, 52) | (33, 1) | (33, 47) | (34, 46) | (2, 2) | (21, 38) | (34, 52) | (22, 37) | (26, 40) |
| Nagano | (44, 10) | (42, 6) | (43, 5) | (41, 5) | (38, 2) | (33, 5) | (34, 1) | (31, 8) | (1, 8) | (28, 50) | (34, 52) | (20, 37) | (15, 40) |
| Gifu | (38, 5) | (33, 6) | (36, 5) | (40, 4) | (36, 52) | (31, 2) | (30, 47) | (31, 46) | (NA, NA) | (19, 37) | (16, 37) | (14, 32) | (12, 33) |
| Shizuoka | (36, 11) | (37, 5) | (38, 6) | (35, 6) | (35, 50) | (30, 1) | (28, 44) | (30, 46) | (NA, NA) | (10, 35) | (27, 52) | (16, 36) | (13, 33) |
| Aichi | (38, 6) | (36, 6) | (36, 5) | (40, 5) | (37, 1) | (28, 52) | (28, 46) | (29, 52) | (2, 2) | (14, 35) | (20, 36) | (16, 33) | (12, 32) |
| Mie | (38, 8) | (42, 4) | (39, 4) | (36, 6) | (34, 5) | (31, 52) | (27, 43) | (32, 52) | (2, 3) | (19, 34) | (22, 40) | (17, 37) | (13, 34) |
| *Kinki* |  |  |  |  |  |  |  |  |  |  |  |  |  |
| Shiga | (36, 9) | (41, 5) | (36, 6) | (41, 6) | (37, 4) | (32, 2) | (32, 2) | (31, 48) | (2, 3) | (15, 30) | (24, 46) | (15, 35) | (11, 36) |
| Kyoto | (36, 52) | (35, 52) | (36, 5) | (37, 2) | (35, 51) | (30, 2) | (29, 45) | (30, 52) | (2, 3) | (13, 36) | (22, 43) | (9, 34) | (10, 33) |
| Osaka | (36, 7) | (35, 52) | (35, 9) | (37, 5) | (35, 5) | (28, 5) | (27, 44) | (28, 52) | (NA, NA) | (7, 36) | (24, 40) | (11, 33) | (8, 32) |
| Hyogo | (39, 4) | (37, 52) | (36, 11) | (40, 4) | (35, 5) | (31, 7) | (28, 52) | (30, 52) | (9, 9) | (12, 38) | (24, 42) | (12, 35) | (12, 38) |
| Nara | (37, 2) | (37, 4) | (38, 8) | (37, 4) | (35, 5) | (30, 2) | (32, 47) | (31, 46) | (2, 2) | (14, 36) | (26, 44) | (14, 32) | (10, 33) |
| Wakayama | (46, 7) | (36, 3) | (46, 13) | (45, 7) | (36, 1) | (27, 3) | (28, 44) | (31, 12) | (1, 12) | (20, 34) | (25, 42) | (13, 32) | (13, 35) |
| *Chugoku* |  |  |  |  |  |  |  |  |  |  |  |  |  |
| Tottori | (39, 52) | (37, 2) | (36, 1) | (40, 1) | (36, 51) | (28, 46) | (31, 44) | (30, 48) | (NA, NA) | (25, 40) | (27, 47) | (20, 38) | (19, 38) |
| Shimane | (36, 5) | (33, 2) | (35, 2) | (34, 6) | (36, 51) | (33, 46) | (30, 41) | (32, 45) | (NA, NA) | (22, 37) | (23, 38) | (20, 35) | (25, 39) |
| Okayama | (37, 6) | (37, 3) | (37, 7) | (35, 4) | (36, 2) | (33, 50) | (30, 43) | (30, 46) | (4, 4) | (19, 38) | (28, 48) | (20, 37) | (16, 39) |
| Hiroshima | (34, 52) | (30, 8) | (35, 6) | (33, 5) | (31, 1) | (30, 50) | (28, 52) | (31, 48) | (6, 6) | (12, 39) | (25, 43) | (15, 35) | (13, 35) |
| Yamaguchi | (35, 11) | (30, 10) | (36, 8) | (32, 11) | (34, 4) | (29, 6) | (23, 43) | (30, 48) | (8, 11) | (14, 40) | (28, 51) | (15, 35) | (12, 36) |
| *Shikoku* |  |  |  |  |  |  |  |  |  |  |  |  |  |
| Tokushima | (39, 6) | (36, 7) | (36, 6) | (36, 9) | (35, 5) | (31, 52) | (27, 43) | (31, 44) | (7, 7) | (23, 37) | (29, 47) | (20, 35) | (14, 34) |
| Kagawa | (37, 3) | (36, 4) | (41, 9) | (40, 4) | (36, 1) | (32, 2) | (31, 43) | (34, 48) | (6, 6) | (21, 38) | (30, 52) | (20, 36) | (13, 33) |
| Ehime | (35, 8) | (35, 5) | (36, 13) | (37, 6) | (36, 52) | (29, 49) | (28, 45) | (31, 45) | (NA, NA) | (22, 40) | (23, 43) | (15, 35) | (13, 37) |
| Kochi | (47, 11) | (40, 6) | (37, 9) | (34, 15) | (37, 3) | (31, 1) | (32, 43) | (31, 43) | (5, 7) | (21, 37) | (28, 48) | (20, 38) | (15, 42) |
| *Kyushu* |  |  |  |  |  |  |  |  |  |  |  |  |  |
| Fukuoka | (18, 46) | (28, 10) | (34, 52) | (46, 16) | (28, 2) | (29, 47) | (26, 44) | (28, 44) | (NA, NA) | (3, 38) | (25, 51) | (10, 34) | (14, 37) |
| Saga | (35, 48) | (33, 6) | (51, 13) | (34, 16) | (37, 52) | (34, 48) | (28, 43) | (31, 44) | (NA, NA) | (3, 29) | (28, 46) | (6, 34) | (14, 34) |
| Nagasaki* | (35, 5) | (33, 6) | (49, 16) | (49, 11) | (33, 1) | (29, 44) | (27, 42) | (29, 43) | (NA, NA) | (3, 31) | (48, 11) | (1, 38) | (19, 36) |
| Kumamoto* | (35, 46) | (34, 6) | (36, 11) | (35, 11) | (33, 1) | (30, 46) | (30, 45) | (28, 44) | (NA, NA) | (3, 28) | (45, 8) | (23, 36) | (15, 33) |
| Oita | (33, 45) | (38, 7) | (36, 9) | (30, 42) | (38, 1) | (30, 50) | (27, 42) | (31, 44) | (NA, NA) | (15, 32) | (24, 42) | (15, 36) | (16, 39) |
| Miyazaki* | (18, 31) | (51, 13) | (36, 52) | (48, 11) | (32, 44) | (31, 45) | (28, 43) | (28, 42) | (NA, NA) | (49, 31) | (49, 18) | (13, 33) | (15, 33) |
| Kagoshima** | (18, 41) | (30, 44) | (33, 44) | (1, 13) | (28, 36) | (28, 46) | (28, 43) | (27, 44) | (34, 10) | (25, 7) | (8, 17) | (8, 36) | (15, 36) |
| Okinawa | (18, 36) | (21, 35) | (18, 35) | (18, 36) | (18, 33) | (18, 35) | (18, 35) | (20, 35) | (38, 53) | (33, 52) | (34, 49) | (16, 33) | (15, 32) |

*Time window is from week 18 to week 17 of the following year by the 2019/2020 season, then from week 45 to week 44 of the following year thereafter

**Time window is from week 18 to week 17 of the following year by the 2022/2023 season, then from week 1 to 52 thereafter

NA: Not applicable

Supplementary Table 4. Gap period analysis: (no gap period introduced)

| **National/prefecture** | **Number of gap period: 0 (no gap period introduced)** | | | | | | | | | | | | | | | |
| --- | --- | --- | --- | --- | --- | --- | --- | --- | --- | --- | --- | --- | --- | --- | --- | --- |
|  | **Median** | **Min** | **Max** | **2012/2013** | **2013/2014** | **2014/2015** | **2015/2016** | **2016/2017** | **2017/2018** | **2018/2019** | **2019/2020** | **2020** | **2021** | **2022** | **2023** | **2024** |
| All | 1 | 0 | 4 | 1 | 2 | 1 | 1 | 1 | 2 | 4 | 1 | 0 | 1 | 2 | 1 | 1 |
| Hokkaido | 1 | 1 | 3 | 1 | 2 | 3 | 1 | 3 | 1 | 3 | 1 | 1 | 1 | 2 | 1 | 3 |
| *Tohoku* |  |  |  |  |  |  |  |  |  |  |  |  |  |  |  |  |
| Aomori | 3 | 1 | 5 | 2 | 4 | 2 | 3 | 2 | 3 | 4 | 5 | 4 | 1 | 2 | 4 | 3 |
| Iwate | 3 | 1 | 6 | 2 | 1 | 4 | 2 | 1 | 2 | 3 | 4 | 6 | 4 | 4 | 4 | 1 |
| Miyagi | 3 | 1 | 5 | 4 | 3 | 2 | 1 | 1 | 3 | 5 | 4 | 3 | 2 | 1 | 3 | 1 |
| Akita | 3 | 2 | 5 | 3 | 2 | 4 | 2 | 2 | 2 | 3 | 2 | 2 | 3 | 5 | 4 | 3 |
| Yamagata | 1 | 1 | 5 | 1 | 1 | 1 | 1 | 1 | 3 | 2 | 5 | 1 | 1 | 2 | 4 | 4 |
| Fukushima | 2 | 0 | 7 | 3 | 2 | 1 | 2 | 1 | 7 | 4 | 3 | 0 | 1 | 3 | 2 | 2 |
| *Kanto* |  |  |  |  |  |  |  |  |  |  |  |  |  |  |  |  |
| Ibaraki | 2 | 1 | 4 | 1 | 1 | 3 | 2 | 1 | 3 | 4 | 3 | 1 | 1 | 1 | 2 | 2 |
| Tochigi | 2 | 1 | 5 | 1 | 2 | 2 | 1 | 1 | 3 | 5 | 3 | 1 | 2 | 2 | 3 | 4 |
| Gunma | 2 | 1 | 4 | 2 | 1 | 1 | 2 | 3 | 4 | 4 | 3 | 1 | 2 | 1 | 2 | 4 |
| Saitama | 2 | 1 | 7 | 1 | 2 | 1 | 2 | 1 | 7 | 6 | 4 | 2 | 1 | 1 | 1 | 3 |
| Chiba | 2 | 1 | 6 | 2 | 3 | 2 | 1 | 4 | 4 | 6 | 4 | 1 | 1 | 2 | 1 | 2 |
| Tokyo | 2 | 0 | 5 | 3 | 2 | 2 | 1 | 2 | 5 | 3 | 2 | 0 | 2 | 1 | 1 | 4 |
| Kanagawa | 3 | 1 | 7 | 3 | 4 | 3 | 2 | 1 | 4 | 7 | 3 | 1 | 2 | 1 | 1 | 4 |
| *Chubu* |  |  |  |  |  |  |  |  |  |  |  |  |  |  |  |  |
| Niigata | 2 | 1 | 6 | 1 | 3 | 3 | 1 | 1 | 4 | 5 | 4 | 2 | 1 | 1 | 6 | 2 |
| Toyama | 2 | 0 | 7 | 1 | 4 | 1 | 1 | 2 | 7 | 7 | 2 | 0 | 3 | 4 | 1 | 1 |
| Ishikawa | 2 | 1 | 6 | 1 | 1 | 3 | 2 | 2 | 5 | 6 | 4 | 1 | 2 | 2 | 3 | 2 |
| Fukui | 4 | 1 | 6 | 4 | 5 | 6 | 2 | 1 | 2 | 5 | 5 | 1 | 1 | 4 | 2 | 4 |
| Yamanashi | 3 | 1 | 6 | 3 | 4 | 4 | 2 | 1 | 2 | 4 | 6 | 2 | 2 | 3 | 4 | 2 |
| Nagano | 2 | 1 | 3 | 2 | 1 | 2 | 1 | 2 | 1 | 3 | 2 | 1 | 2 | 2 | 2 | 1 |
| Gifu | 2 | 0 | 7 | 1 | 1 | 1 | 2 | 3 | 2 | 7 | 1 | 0 | 2 | 2 | 3 | 2 |
| Shizuoka | 1 | 0 | 5 | 1 | 1 | 1 | 1 | 2 | 2 | 4 | 2 | 0 | 1 | 1 | 5 | 3 |
| Aichi | 1 | 1 | 5 | 1 | 3 | 1 | 1 | 1 | 3 | 5 | 2 | 1 | 1 | 3 | 1 | 1 |
| Mie | 2 | 1 | 5 | 2 | 3 | 1 | 1 | 2 | 3 | 5 | 3 | 2 | 1 | 2 | 1 | 2 |
| *Kinki* |  |  |  |  |  |  |  |  |  |  |  |  |  |  |  |  |
| Shiga | 2 | 1 | 5 | 2 | 3 | 2 | 2 | 4 | 4 | 5 | 3 | 1 | 2 | 1 | 1 | 2 |
| Kyoto | 2 | 1 | 7 | 3 | 3 | 1 | 1 | 2 | 2 | 7 | 2 | 1 | 2 | 3 | 1 | 4 |
| Osaka | 1 | 0 | 5 | 1 | 4 | 1 | 1 | 2 | 3 | 3 | 1 | 0 | 1 | 1 | 1 | 5 |
| Hyogo | 1 | 1 | 3 | 1 | 2 | 1 | 1 | 1 | 1 | 2 | 2 | 1 | 2 | 3 | 2 | 1 |
| Nara | 3 | 1 | 6 | 4 | 2 | 3 | 1 | 5 | 4 | 6 | 6 | 2 | 1 | 1 | 2 | 4 |
| Wakayama | 3 | 1 | 4 | 3 | 2 | 4 | 2 | 3 | 1 | 4 | 2 | 1 | 3 | 1 | 3 | 3 |
| *Chugoku* |  |  |  |  |  |  |  |  |  |  |  |  |  |  |  |  |
| Tottori | 2 | 0 | 5 | 2 | 2 | 1 | 3 | 2 | 5 | 5 | 1 | 0 | 1 | 1 | 2 | 2 |
| Shimane | 3 | 0 | 6 | 1 | 4 | 3 | 1 | 3 | 3 | 6 | 1 | 0 | 2 | 4 | 2 | 3 |
| Okayama | 2 | 1 | 6 | 1 | 2 | 1 | 2 | 2 | 4 | 6 | 3 | 2 | 2 | 2 | 4 | 1 |
| Hiroshima | 2 | 1 | 5 | 5 | 1 | 1 | 1 | 2 | 4 | 5 | 2 | 1 | 3 | 3 | 2 | 1 |
| Yamaguchi | 1 | 1 | 5 | 1 | 1 | 1 | 2 | 3 | 2 | 5 | 4 | 2 | 1 | 1 | 1 | 1 |
| *Shikoku* |  |  |  |  |  |  |  |  |  |  |  |  |  |  |  |  |
| Tokushima | 1 | 1 | 7 | 2 | 1 | 1 | 1 | 1 | 2 | 7 | 2 | 1 | 1 | 1 | 1 | 2 |
| Kagawa | 2 | 1 | 6 | 2 | 2 | 2 | 2 | 2 | 3 | 6 | 4 | 1 | 1 | 1 | 4 | 3 |
| Ehime | 2 | 0 | 6 | 2 | 2 | 2 | 2 | 3 | 4 | 3 | 1 | 0 | 1 | 6 | 4 | 3 |
| Kochi | 2 | 1 | 5 | 3 | 2 | 2 | 1 | 4 | 2 | 4 | 5 | 3 | 1 | 1 | 4 | 1 |
| *Kyushu* |  |  |  |  |  |  |  |  |  |  |  |  |  |  |  |  |
| Fukuoka | 2 | 0 | 5 | 4 | 1 | 4 | 2 | 2 | 5 | 4 | 1 | 0 | 2 | 2 | 2 | 1 |
| Saga | 3 | 0 | 7 | 5 | 2 | 3 | 2 | 3 | 6 | 7 | 1 | 0 | 2 | 4 | 3 | 2 |
| Nagasaki* | 2 | 0 | 5 | 1 | 2 | 2 | 5 | 3 | 3 | 3 | 1 | 0 | 2 | 4 | 3 | 2 |
| Kumamoto* | 3 | 0 | 8 | 4 | 2 | 1 | 1 | 2 | 3 | 4 | 1 | 0 | 3 | 3 | 8 | 3 |
| Oita | 2 | 0 | 6 | 4 | 1 | 2 | 5 | 2 | 3 | 6 | 1 | 0 | 1 | 2 | 1 | 2 |
| Miyazaki* | 3 | 0 | 8 | 6 | 2 | 3 | 3 | 7 | 6 | 1 | 1 | 0 | 2 | 8 | 4 | 2 |
| Kagoshima** | 3 | 1 | 8 | 2 | 4 | 4 | 4 | 8 | 5 | 3 | 2 | 2 | 1 | 5 | 2 | 3 |
| Okinawa | 3 | 1 | 4 | 4 | 4 | 3 | 4 | 2 | 4 | 1 | 3 | 2 | 3 | 2 | 1 | 1 |

*Time window is from week 18 to week 17 of the following year by the 2019/2020 season, then from week 45 to week 44 of the following year thereafter

**Time window is from week 18 to week 17 of the following year by the 2022/2023 season, then from week 1 to 52 thereafter.

**(Continued)**

| **National/prefecture** | **Number of gap period: 1 week** | | | | | | | | | | | | | | | |
| --- | --- | --- | --- | --- | --- | --- | --- | --- | --- | --- | --- | --- | --- | --- | --- | --- |
|  | **Median** | **Min** | **Max** | **2012/2013** | **2013/2014** | **2014/2015** | **2015/2016** | **2016/2017** | **2017/2018** | **2018/2019** | **2019/2020** | **2020** | **2021** | **2022** | **2023** | **2024** |
| All | 1 | 0 | 2 | 1 | 1 | 1 | 1 | 1 | 2 | 2 | 1 | 0 | 1 | 2 | 1 | 1 |
| Hokkaido | 1 | 1 | 3 | 1 | 2 | 2 | 1 | 2 | 1 | 3 | 1 | 1 | 1 | 2 | 1 | 2 |
| *Tohoku* |  |  |  |  |  |  |  |  |  |  |  |  |  |  |  |  |
| Aomori | 3 | 1 | 4 | 1 | 4 | 2 | 2 | 1 | 3 | 3 | 4 | 3 | 1 | 1 | 4 | 3 |
| Iwate | 2 | 1 | 5 | 2 | 1 | 2 | 1 | 1 | 1 | 2 | 2 | 5 | 2 | 2 | 2 | 1 |
| Miyagi | 2 | 1 | 4 | 4 | 2 | 2 | 1 | 1 | 2 | 2 | 2 | 1 | 1 | 1 | 3 | 1 |
| Akita | 2 | 1 | 3 | 3 | 2 | 2 | 2 | 1 | 1 | 2 | 1 | 1 | 3 | 2 | 3 | 2 |
| Yamagata | 1 | 1 | 4 | 1 | 1 | 1 | 1 | 1 | 3 | 1 | 4 | 1 | 1 | 1 | 2 | 3 |
| Fukushima | 1 | 0 | 3 | 2 | 1 | 1 | 1 | 1 | 2 | 3 | 2 | 0 | 1 | 2 | 1 | 2 |
| *Kanto* |  |  |  |  |  |  |  |  |  |  |  |  |  |  |  |  |
| Ibaraki | 1 | 1 | 3 | 1 | 1 | 2 | 1 | 1 | 3 | 3 | 2 | 1 | 1 | 1 | 2 | 2 |
| Tochigi | 1 | 1 | 5 | 1 | 1 | 1 | 1 | 1 | 2 | 5 | 3 | 1 | 2 | 2 | 1 | 2 |
| Gunma | 2 | 1 | 4 | 2 | 1 | 1 | 1 | 2 | 3 | 4 | 3 | 1 | 2 | 1 | 2 | 4 |
| Saitama | 1 | 1 | 4 | 1 | 2 | 1 | 1 | 1 | 1 | 4 | 3 | 1 | 1 | 1 | 1 | 3 |
| Chiba | 1 | 1 | 3 | 1 | 1 | 2 | 1 | 2 | 1 | 3 | 3 | 1 | 1 | 1 | 1 | 2 |
| Tokyo | 1 | 0 | 5 | 1 | 1 | 1 | 1 | 1 | 5 | 3 | 1 | 0 | 1 | 1 | 1 | 3 |
| Kanagawa | 1 | 1 | 4 | 1 | 2 | 1 | 1 | 1 | 2 | 4 | 2 | 1 | 1 | 1 | 1 | 4 |
| *Chubu* |  |  |  |  |  |  |  |  |  |  |  |  |  |  |  |  |
| Niigata | 2 | 1 | 3 | 1 | 2 | 3 | 1 | 1 | 3 | 3 | 3 | 2 | 1 | 1 | 2 | 1 |
| Toyama | 1 | 0 | 5 | 1 | 1 | 1 | 1 | 2 | 3 | 5 | 1 | 0 | 3 | 3 | 1 | 1 |
| Ishikawa | 2 | 1 | 4 | 1 | 1 | 3 | 1 | 2 | 4 | 3 | 3 | 1 | 2 | 1 | 2 | 1 |
| Fukui | 2 | 1 | 4 | 3 | 2 | 2 | 1 | 1 | 2 | 3 | 4 | 1 | 1 | 4 | 1 | 4 |
| Yamanashi | 2 | 1 | 3 | 1 | 2 | 2 | 2 | 1 | 1 | 3 | 3 | 2 | 1 | 2 | 2 | 2 |
| Nagano | 1 | 1 | 3 | 1 | 1 | 1 | 1 | 1 | 1 | 3 | 2 | 1 | 2 | 1 | 2 | 1 |
| Gifu | 1 | 0 | 4 | 1 | 1 | 1 | 1 | 2 | 2 | 4 | 1 | 0 | 1 | 2 | 1 | 2 |
| Shizuoka | 1 | 0 | 5 | 1 | 1 | 1 | 1 | 2 | 2 | 2 | 2 | 0 | 1 | 1 | 5 | 2 |
| Aichi | 1 | 1 | 3 | 1 | 2 | 1 | 1 | 1 | 1 | 3 | 1 | 1 | 1 | 3 | 1 | 1 |
| Mie | 1 | 1 | 2 | 1 | 2 | 1 | 1 | 1 | 1 | 2 | 2 | 2 | 1 | 1 | 1 | 1 |
| *Kinki* |  |  |  |  |  |  |  |  |  |  |  |  |  |  |  |  |
| Shiga | 2 | 1 | 4 | 1 | 3 | 1 | 2 | 3 | 3 | 4 | 2 | 1 | 1 | 1 | 1 | 2 |
| Kyoto | 1 | 1 | 3 | 2 | 1 | 1 | 1 | 2 | 1 | 3 | 1 | 1 | 2 | 2 | 1 | 3 |
| Osaka | 1 | 0 | 4 | 1 | 1 | 1 | 1 | 1 | 2 | 2 | 1 | 0 | 1 | 1 | 1 | 4 |
| Hyogo | 1 | 1 | 2 | 1 | 1 | 1 | 1 | 1 | 1 | 1 | 2 | 1 | 1 | 1 | 2 | 1 |
| Nara | 2 | 1 | 4 | 3 | 2 | 1 | 1 | 2 | 3 | 3 | 3 | 2 | 1 | 1 | 1 | 4 |
| Wakayama | 1 | 1 | 2 | 1 | 1 | 2 | 1 | 1 | 1 | 2 | 2 | 1 | 2 | 1 | 2 | 2 |
| *Chugoku* |  |  |  |  |  |  |  |  |  |  |  |  |  |  |  |  |
| Tottori | 1 | 0 | 4 | 1 | 1 | 1 | 1 | 1 | 2 | 4 | 1 | 0 | 1 | 1 | 2 | 1 |
| Shimane | 2 | 0 | 3 | 1 | 3 | 2 | 1 | 2 | 1 | 3 | 1 | 0 | 2 | 2 | 2 | 2 |
| Okayama | 2 | 1 | 5 | 1 | 2 | 1 | 2 | 1 | 1 | 5 | 2 | 2 | 1 | 2 | 3 | 1 |
| Hiroshima | 1 | 1 | 3 | 2 | 1 | 1 | 1 | 1 | 2 | 3 | 2 | 1 | 3 | 2 | 1 | 1 |
| Yamaguchi | 1 | 1 | 2 | 1 | 1 | 1 | 2 | 2 | 2 | 1 | 2 | 1 | 1 | 1 | 1 | 1 |
| *Shikoku* |  |  |  |  |  |  |  |  |  |  |  |  |  |  |  |  |
| Tokushima | 1 | 1 | 3 | 2 | 1 | 1 | 1 | 1 | 2 | 3 | 2 | 1 | 1 | 1 | 1 | 1 |
| Kagawa | 1 | 1 | 6 | 1 | 1 | 1 | 1 | 2 | 3 | 6 | 3 | 1 | 1 | 1 | 3 | 2 |
| Ehime | 2 | 0 | 5 | 2 | 1 | 2 | 2 | 2 | 4 | 3 | 1 | 0 | 1 | 5 | 3 | 3 |
| Kochi | 1 | 1 | 5 | 1 | 1 | 2 | 1 | 1 | 2 | 3 | 5 | 3 | 1 | 1 | 4 | 1 |
| *Kyushu* |  |  |  |  |  |  |  |  |  |  |  |  |  |  |  |  |
| Fukuoka | 1 | 0 | 3 | 3 | 1 | 2 | 1 | 1 | 3 | 3 | 1 | 0 | 2 | 2 | 1 | 1 |
| Saga | 2 | 0 | 4 | 3 | 1 | 2 | 2 | 2 | 4 | 3 | 1 | 0 | 2 | 3 | 1 | 1 |
| Nagasaki* | 1 | 0 | 3 | 1 | 1 | 2 | 3 | 3 | 3 | 2 | 1 | 0 | 1 | 3 | 1 | 1 |
| Kumamoto* | 1 | 0 | 4 | 1 | 1 | 1 | 1 | 1 | 3 | 4 | 1 | 0 | 2 | 3 | 2 | 1 |
| Oita | 1 | 0 | 4 | 1 | 1 | 2 | 3 | 2 | 2 | 4 | 1 | 0 | 1 | 2 | 1 | 1 |
| Miyazaki* | 2 | 0 | 4 | 4 | 2 | 1 | 2 | 4 | 1 | 1 | 1 | 0 | 2 | 3 | 3 | 1 |
| Kagoshima** | 2 | 1 | 5 | 2 | 2 | 3 | 3 | 4 | 4 | 3 | 2 | 1 | 1 | 5 | 2 | 2 |
| Okinawa | 2 | 1 | 3 | 2 | 3 | 2 | 3 | 2 | 2 | 1 | 2 | 2 | 2 | 2 | 1 | 1 |

*Time window is from week 18 to week 17 of the following year by the 2019/2020 season, then from week 45 to week 44 of the following year thereafter

**Time window is from week 18 to week 17 of the following year by the 2022/2023 season, then from week 1 to 52 thereafter.

**(Continued)**

| **National/prefecture** | **Number of gap period: 2 weeks** | | | | | | | | | | | | | | | |
| --- | --- | --- | --- | --- | --- | --- | --- | --- | --- | --- | --- | --- | --- | --- | --- | --- |
|  | **Median** | **Min** | **Max** | **2012/2013** | **2013/2014** | **2014/2015** | **2015/2016** | **2016/2017** | **2017/2018** | **2018/2019** | **2019/2020** | **2020** | **2021** | **2022** | **2023** | **2024** |
| All | 1 | 0 | 2 | 1 | 1 | 1 | 1 | 1 | 2 | 2 | 1 | 0 | 1 | 2 | 1 | 1 |
| Hokkaido | 1 | 1 | 2 | 1 | 2 | 1 | 1 | 2 | 1 | 2 | 1 | 1 | 1 | 2 | 1 | 1 |
| *Tohoku* |  |  |  |  |  |  |  |  |  |  |  |  |  |  |  |  |
| Aomori | 2 | 1 | 3 | 1 | 3 | 2 | 1 | 1 | 2 | 2 | 3 | 2 | 1 | 1 | 2 | 3 |
| Iwate | 1 | 1 | 3 | 2 | 1 | 1 | 1 | 1 | 1 | 1 | 2 | 3 | 1 | 2 | 1 | 1 |
| Miyagi | 1 | 1 | 2 | 2 | 1 | 2 | 1 | 1 | 2 | 2 | 2 | 1 | 1 | 1 | 2 | 1 |
| Akita | 2 | 1 | 3 | 3 | 1 | 2 | 2 | 1 | 1 | 2 | 1 | 1 | 3 | 2 | 3 | 2 |
| Yamagata | 1 | 1 | 4 | 1 | 1 | 1 | 1 | 1 | 2 | 1 | 4 | 1 | 1 | 1 | 2 | 1 |
| Fukushima | 1 | 0 | 2 | 2 | 1 | 1 | 1 | 1 | 1 | 2 | 2 | 0 | 1 | 2 | 1 | 2 |
| *Kanto* |  |  |  |  |  |  |  |  |  |  |  |  |  |  |  |  |
| Ibaraki | 1 | 1 | 3 | 1 | 1 | 1 | 1 | 1 | 3 | 2 | 2 | 1 | 1 | 1 | 2 | 2 |
| Tochigi | 1 | 1 | 3 | 1 | 1 | 1 | 1 | 1 | 1 | 3 | 3 | 1 | 1 | 2 | 1 | 2 |
| Gunma | 2 | 1 | 3 | 2 | 1 | 1 | 1 | 2 | 1 | 3 | 2 | 1 | 2 | 1 | 2 | 3 |
| Saitama | 1 | 1 | 3 | 1 | 1 | 1 | 1 | 1 | 1 | 3 | 3 | 1 | 1 | 1 | 1 | 3 |
| Chiba | 1 | 1 | 3 | 1 | 1 | 2 | 1 | 1 | 1 | 2 | 3 | 1 | 1 | 1 | 1 | 2 |
| Tokyo | 1 | 0 | 4 | 1 | 1 | 1 | 1 | 1 | 4 | 2 | 1 | 0 | 1 | 1 | 1 | 3 |
| Kanagawa | 1 | 1 | 4 | 1 | 1 | 1 | 1 | 1 | 2 | 4 | 2 | 1 | 1 | 1 | 1 | 3 |
| *Chubu* |  |  |  |  |  |  |  |  |  |  |  |  |  |  |  |  |
| Niigata | 1 | 1 | 3 | 1 | 2 | 3 | 1 | 1 | 2 | 2 | 3 | 2 | 1 | 1 | 1 | 1 |
| Toyama | 1 | 0 | 4 | 1 | 1 | 1 | 1 | 2 | 3 | 4 | 1 | 0 | 2 | 3 | 1 | 1 |
| Ishikawa | 1 | 1 | 3 | 1 | 1 | 2 | 1 | 2 | 3 | 3 | 3 | 1 | 2 | 1 | 1 | 1 |
| Fukui | 2 | 1 | 4 | 2 | 1 | 2 | 1 | 1 | 2 | 3 | 4 | 1 | 1 | 3 | 1 | 2 |
| Yamanashi | 2 | 1 | 3 | 1 | 2 | 1 | 1 | 1 | 1 | 3 | 3 | 2 | 1 | 2 | 2 | 2 |
| Nagano | 1 | 1 | 2 | 1 | 1 | 1 | 1 | 1 | 1 | 2 | 2 | 1 | 1 | 1 | 2 | 1 |
| Gifu | 1 | 0 | 4 | 1 | 1 | 1 | 1 | 2 | 1 | 4 | 1 | 0 | 1 | 1 | 1 | 1 |
| Shizuoka | 1 | 0 | 2 | 1 | 1 | 1 | 1 | 1 | 2 | 2 | 1 | 0 | 1 | 1 | 2 | 1 |
| Aichi | 1 | 1 | 3 | 1 | 1 | 1 | 1 | 1 | 1 | 3 | 1 | 1 | 1 | 2 | 1 | 1 |
| Mie | 1 | 1 | 2 | 1 | 2 | 1 | 1 | 1 | 1 | 1 | 1 | 1 | 1 | 1 | 1 | 1 |
| *Kinki* |  |  |  |  |  |  |  |  |  |  |  |  |  |  |  |  |
| Shiga | 1 | 1 | 3 | 1 | 2 | 1 | 2 | 2 | 3 | 2 | 1 | 1 | 1 | 1 | 1 | 1 |
| Kyoto | 1 | 1 | 3 | 1 | 1 | 1 | 1 | 2 | 1 | 3 | 1 | 1 | 2 | 2 | 1 | 3 |
| Osaka | 1 | 0 | 2 | 1 | 1 | 1 | 1 | 1 | 1 | 2 | 1 | 0 | 1 | 1 | 1 | 1 |
| Hyogo | 1 | 1 | 2 | 1 | 1 | 1 | 1 | 1 | 1 | 1 | 2 | 1 | 1 | 1 | 2 | 1 |
| Nara | 2 | 1 | 3 | 2 | 1 | 1 | 1 | 2 | 3 | 3 | 3 | 2 | 1 | 1 | 1 | 3 |
| Wakayama | 1 | 1 | 2 | 1 | 1 | 2 | 1 | 1 | 1 | 2 | 2 | 1 | 1 | 1 | 2 | 2 |
| *Chugoku* |  |  |  |  |  |  |  |  |  |  |  |  |  |  |  |  |
| Tottori | 1 | 0 | 4 | 1 | 1 | 1 | 1 | 1 | 2 | 4 | 1 | 0 | 1 | 1 | 1 | 1 |
| Shimane | 1 | 0 | 2 | 1 | 2 | 1 | 1 | 1 | 1 | 2 | 1 | 0 | 1 | 1 | 2 | 2 |
| Okayama | 1 | 1 | 3 | 1 | 1 | 1 | 1 | 1 | 1 | 3 | 2 | 2 | 1 | 2 | 2 | 1 |
| Hiroshima | 1 | 1 | 2 | 2 | 1 | 1 | 1 | 1 | 2 | 2 | 2 | 1 | 2 | 2 | 1 | 1 |
| Yamaguchi | 1 | 1 | 2 | 1 | 1 | 1 | 1 | 2 | 1 | 1 | 2 | 1 | 1 | 1 | 1 | 1 |
| *Shikoku* |  |  |  |  |  |  |  |  |  |  |  |  |  |  |  |  |
| Tokushima | 1 | 1 | 2 | 1 | 1 | 1 | 1 | 1 | 2 | 2 | 2 | 1 | 1 | 1 | 1 | 1 |
| Kagawa | 1 | 1 | 5 | 1 | 1 | 1 | 1 | 2 | 3 | 5 | 3 | 1 | 1 | 1 | 2 | 2 |
| Ehime | 2 | 0 | 4 | 2 | 1 | 1 | 2 | 2 | 4 | 3 | 1 | 0 | 1 | 2 | 3 | 3 |
| Kochi | 1 | 1 | 3 | 1 | 1 | 1 | 1 | 1 | 2 | 3 | 3 | 2 | 1 | 1 | 3 | 1 |
| *Kyushu* |  |  |  |  |  |  |  |  |  |  |  |  |  |  |  |  |
| Fukuoka | 1 | 0 | 3 | 3 | 1 | 1 | 1 | 1 | 3 | 3 | 1 | 0 | 2 | 2 | 1 | 1 |
| Saga | 1 | 0 | 3 | 3 | 1 | 2 | 1 | 2 | 1 | 2 | 1 | 0 | 2 | 2 | 1 | 1 |
| Nagasaki* | 1 | 0 | 3 | 1 | 1 | 2 | 1 | 2 | 3 | 2 | 1 | 0 | 1 | 3 | 1 | 1 |
| Kumamoto* | 1 | 0 | 3 | 1 | 1 | 1 | 1 | 1 | 3 | 2 | 1 | 0 | 2 | 2 | 1 | 1 |
| Oita | 1 | 0 | 4 | 1 | 1 | 1 | 2 | 2 | 2 | 4 | 1 | 0 | 1 | 2 | 1 | 1 |
| Miyazaki* | 1 | 0 | 4 | 3 | 2 | 1 | 1 | 4 | 1 | 1 | 1 | 0 | 2 | 1 | 3 | 1 |
| Kagoshima** | 2 | 1 | 3 | 2 | 2 | 2 | 3 | 3 | 2 | 2 | 2 | 1 | 1 | 3 | 1 | 2 |
| Okinawa | 2 | 1 | 3 | 2 | 2 | 2 | 3 | 2 | 2 | 1 | 1 | 2 | 2 | 2 | 1 | 1 |

*Time window is from week 18 to week 17 of the following year by the 2019/2020 season, then from week 45 to week 44 of the following year thereafter

**Time window is from week 18 to week 17 of the following year by the 2022/2023 season, then from week 1 to 52 thereafter.

**(Continued)**

| **National/prefecture** | **Number of gap period: 3 weeks** | | | | | | | | | | | | | | | |
| --- | --- | --- | --- | --- | --- | --- | --- | --- | --- | --- | --- | --- | --- | --- | --- | --- |
|  | **Median** | **Min** | **Max** | **2012/2013** | **2013/2014** | **2014/2015** | **2015/2016** | **2016/2017** | **2017/2018** | **2018/2019** | **2019/2020** | **2020** | **2021** | **2022** | **2023** | **2024** |
| All | 1 | 0 | 2 | 1 | 1 | 1 | 1 | 1 | 2 | 1 | 1 | 0 | 1 | 2 | 1 | 1 |
| Hokkaido | 1 | 1 | 2 | 1 | 2 | 1 | 1 | 2 | 1 | 2 | 1 | 1 | 1 | 2 | 1 | 1 |
| *Tohoku* |  |  |  |  |  |  |  |  |  |  |  |  |  |  |  |  |
| Aomori | 2 | 1 | 3 | 1 | 2 | 2 | 1 | 1 | 2 | 2 | 3 | 2 | 1 | 1 | 1 | 2 |
| Iwate | 1 | 1 | 3 | 1 | 1 | 1 | 1 | 1 | 1 | 1 | 2 | 3 | 1 | 2 | 1 | 1 |
| Miyagi | 1 | 1 | 2 | 2 | 1 | 1 | 1 | 1 | 1 | 2 | 1 | 1 | 1 | 1 | 2 | 1 |
| Akita | 2 | 1 | 2 | 2 | 1 | 2 | 2 | 1 | 1 | 2 | 1 | 1 | 2 | 2 | 2 | 2 |
| Yamagata | 1 | 1 | 2 | 1 | 1 | 1 | 1 | 1 | 2 | 1 | 2 | 1 | 1 | 1 | 2 | 1 |
| Fukushima | 1 | 0 | 2 | 1 | 1 | 1 | 1 | 1 | 1 | 2 | 1 | 0 | 1 | 1 | 1 | 2 |
| *Kanto* |  |  |  |  |  |  |  |  |  |  |  |  |  |  |  |  |
| Ibaraki | 1 | 1 | 2 | 1 | 1 | 1 | 1 | 1 | 2 | 1 | 2 | 1 | 1 | 1 | 2 | 2 |
| Tochigi | 1 | 1 | 3 | 1 | 1 | 1 | 1 | 1 | 1 | 2 | 3 | 1 | 1 | 2 | 1 | 2 |
| Gunma | 1 | 1 | 2 | 2 | 1 | 1 | 1 | 2 | 1 | 2 | 1 | 1 | 1 | 1 | 2 | 2 |
| Saitama | 1 | 1 | 3 | 1 | 1 | 1 | 1 | 1 | 1 | 3 | 2 | 1 | 1 | 1 | 1 | 2 |
| Chiba | 1 | 1 | 3 | 1 | 1 | 2 | 1 | 1 | 1 | 2 | 3 | 1 | 1 | 1 | 1 | 2 |
| Tokyo | 1 | 0 | 3 | 1 | 1 | 1 | 1 | 1 | 2 | 2 | 1 | 0 | 1 | 1 | 1 | 3 |
| Kanagawa | 1 | 1 | 4 | 1 | 1 | 1 | 1 | 1 | 2 | 4 | 2 | 1 | 1 | 1 | 1 | 3 |
| *Chubu* |  |  |  |  |  |  |  |  |  |  |  |  |  |  |  |  |
| Niigata | 1 | 1 | 3 | 1 | 2 | 3 | 1 | 1 | 2 | 2 | 3 | 2 | 1 | 1 | 1 | 1 |
| Toyama | 1 | 0 | 4 | 1 | 1 | 1 | 1 | 2 | 2 | 4 | 1 | 0 | 2 | 3 | 1 | 1 |
| Ishikawa | 1 | 1 | 3 | 1 | 1 | 2 | 1 | 2 | 3 | 2 | 3 | 1 | 2 | 1 | 1 | 1 |
| Fukui | 1 | 1 | 3 | 1 | 1 | 2 | 1 | 1 | 2 | 2 | 3 | 1 | 1 | 3 | 1 | 2 |
| Yamanashi | 1 | 1 | 3 | 1 | 1 | 1 | 1 | 1 | 1 | 2 | 3 | 2 | 1 | 2 | 2 | 1 |
| Nagano | 1 | 1 | 2 | 1 | 1 | 1 | 1 | 1 | 1 | 2 | 2 | 1 | 1 | 1 | 2 | 1 |
| Gifu | 1 | 0 | 2 | 1 | 1 | 1 | 1 | 2 | 1 | 2 | 1 | 0 | 1 | 1 | 1 | 1 |
| Shizuoka | 1 | 0 | 2 | 1 | 1 | 1 | 1 | 1 | 2 | 2 | 1 | 0 | 1 | 1 | 1 | 1 |
| Aichi | 1 | 1 | 3 | 1 | 1 | 1 | 1 | 1 | 1 | 3 | 1 | 1 | 1 | 2 | 1 | 1 |
| Mie | 1 | 1 | 2 | 1 | 2 | 1 | 1 | 1 | 1 | 1 | 1 | 1 | 1 | 1 | 1 | 1 |
| *Kinki* |  |  |  |  |  |  |  |  |  |  |  |  |  |  |  |  |
| Shiga | 1 | 1 | 2 | 1 | 1 | 1 | 1 | 1 | 2 | 2 | 1 | 1 | 1 | 1 | 1 | 1 |
| Kyoto | 1 | 1 | 3 | 1 | 1 | 1 | 1 | 2 | 1 | 2 | 1 | 1 | 2 | 2 | 1 | 3 |
| Osaka | 1 | 0 | 2 | 1 | 1 | 1 | 1 | 1 | 1 | 2 | 1 | 0 | 1 | 1 | 1 | 1 |
| Hyogo | 1 | 1 | 2 | 1 | 1 | 1 | 1 | 1 | 1 | 1 | 2 | 1 | 1 | 1 | 2 | 1 |
| Nara | 1 | 1 | 3 | 1 | 1 | 1 | 1 | 1 | 1 | 1 | 3 | 2 | 1 | 1 | 1 | 2 |
| Wakayama | 1 | 1 | 2 | 1 | 1 | 1 | 1 | 1 | 1 | 2 | 2 | 1 | 1 | 1 | 2 | 1 |
| *Chugoku* |  |  |  |  |  |  |  |  |  |  |  |  |  |  |  |  |
| Tottori | 1 | 0 | 3 | 1 | 1 | 1 | 1 | 1 | 2 | 3 | 1 | 0 | 1 | 1 | 1 | 1 |
| Shimane | 1 | 0 | 2 | 1 | 2 | 1 | 1 | 1 | 1 | 2 | 1 | 0 | 1 | 1 | 2 | 2 |
| Okayama | 1 | 1 | 3 | 1 | 1 | 1 | 1 | 1 | 1 | 3 | 2 | 2 | 1 | 2 | 2 | 1 |
| Hiroshima | 1 | 1 | 2 | 1 | 1 | 1 | 1 | 1 | 2 | 1 | 2 | 1 | 2 | 2 | 1 | 1 |
| Yamaguchi | 1 | 1 | 2 | 1 | 1 | 1 | 1 | 2 | 1 | 1 | 1 | 1 | 1 | 1 | 1 | 1 |
| *Shikoku* |  |  |  |  |  |  |  |  |  |  |  |  |  |  |  |  |
| Tokushima | 1 | 1 | 2 | 1 | 1 | 1 | 1 | 1 | 2 | 2 | 2 | 1 | 1 | 1 | 1 | 1 |
| Kagawa | 1 | 1 | 4 | 1 | 1 | 1 | 1 | 1 | 2 | 4 | 3 | 1 | 1 | 1 | 2 | 1 |
| Ehime | 2 | 0 | 3 | 2 | 1 | 1 | 1 | 2 | 3 | 3 | 1 | 0 | 1 | 2 | 3 | 3 |
| Kochi | 1 | 1 | 3 | 1 | 1 | 1 | 1 | 1 | 1 | 3 | 2 | 1 | 1 | 1 | 3 | 1 |
| *Kyushu* |  |  |  |  |  |  |  |  |  |  |  |  |  |  |  |  |
| Fukuoka | 1 | 0 | 3 | 3 | 1 | 1 | 1 | 1 | 2 | 2 | 1 | 0 | 2 | 2 | 1 | 1 |
| Saga | 1 | 0 | 2 | 2 | 1 | 1 | 1 | 2 | 1 | 2 | 1 | 0 | 1 | 2 | 1 | 1 |
| Nagasaki* | 1 | 0 | 3 | 1 | 1 | 2 | 1 | 2 | 3 | 2 | 1 | 0 | 1 | 3 | 1 | 1 |
| Kumamoto* | 1 | 0 | 3 | 1 | 1 | 1 | 1 | 1 | 3 | 1 | 1 | 0 | 1 | 2 | 1 | 1 |
| Oita | 1 | 0 | 3 | 1 | 1 | 1 | 2 | 1 | 1 | 3 | 1 | 0 | 1 | 2 | 1 | 1 |
| Miyazaki* | 1 | 0 | 3 | 2 | 2 | 1 | 1 | 3 | 1 | 1 | 1 | 0 | 2 | 1 | 3 | 1 |
| Kagoshima** | 2 | 1 | 3 | 2 | 2 | 2 | 3 | 3 | 2 | 2 | 2 | 1 | 1 | 3 | 1 | 2 |
| Okinawa | 2 | 1 | 3 | 2 | 2 | 2 | 3 | 2 | 2 | 1 | 1 | 2 | 2 | 2 | 1 | 1 |

*Time window is from week 18 to week 17 of the following year by the 2019/2020 season, then from week 45 to week 44 of the following year thereafter

**Time window is from week 18 to week 17 of the following year by the 2022/2023 season, then from week 1 to 52 thereafter.

**(Continued)**

| **National/prefecture** | **Number of gap period: 4 weeks** | | | | | | | | | | | | | | | |
| --- | --- | --- | --- | --- | --- | --- | --- | --- | --- | --- | --- | --- | --- | --- | --- | --- |
|  | **Median** | **Min** | **Max** | **2012/2013** | **2013/2014** | **2014/2015** | **2015/2016** | **2016/2017** | **2017/2018** | **2018/2019** | **2019/2020** | **2020** | **2021** | **2022** | **2023** | **2024** |
| All | 1 | 0 | 2 | 1 | 1 | 1 | 1 | 1 | 2 | 1 | 1 | 0 | 1 | 2 | 1 | 1 |
| Hokkaido | 1 | 1 | 2 | 1 | 1 | 1 | 1 | 2 | 1 | 1 | 1 | 1 | 1 | 2 | 1 | 1 |
| *Tohoku* |  |  |  |  |  |  |  |  |  |  |  |  |  |  |  |  |
| Aomori | 1 | 1 | 2 | 1 | 2 | 1 | 1 | 1 | 2 | 2 | 2 | 2 | 1 | 1 | 1 | 2 |
| Iwate | 1 | 1 | 2 | 1 | 1 | 1 | 1 | 1 | 1 | 1 | 1 | 2 | 1 | 2 | 1 | 1 |
| Miyagi | 1 | 1 | 2 | 1 | 1 | 1 | 1 | 1 | 1 | 2 | 1 | 1 | 1 | 1 | 2 | 1 |
| Akita | 1 | 1 | 2 | 2 | 1 | 2 | 1 | 1 | 1 | 1 | 1 | 1 | 2 | 2 | 2 | 2 |
| Yamagata | 1 | 1 | 2 | 1 | 1 | 1 | 1 | 1 | 2 | 1 | 2 | 1 | 1 | 1 | 2 | 1 |
| Fukushima | 1 | 0 | 2 | 1 | 1 | 1 | 1 | 1 | 1 | 2 | 1 | 0 | 1 | 1 | 1 | 2 |
| *Kanto* |  |  |  |  |  |  |  |  |  |  |  |  |  |  |  |  |
| Ibaraki | 1 | 1 | 2 | 1 | 1 | 1 | 1 | 1 | 2 | 1 | 2 | 1 | 1 | 1 | 2 | 2 |
| Tochigi | 1 | 1 | 3 | 1 | 1 | 1 | 1 | 1 | 1 | 2 | 3 | 1 | 1 | 2 | 1 | 1 |
| Gunma | 1 | 1 | 2 | 2 | 1 | 1 | 1 | 1 | 1 | 2 | 1 | 1 | 1 | 1 | 2 | 2 |
| Saitama | 1 | 1 | 2 | 1 | 1 | 1 | 1 | 1 | 1 | 1 | 2 | 1 | 1 | 1 | 1 | 2 |
| Chiba | 1 | 1 | 3 | 1 | 1 | 1 | 1 | 1 | 1 | 2 | 3 | 1 | 1 | 1 | 1 | 2 |
| Tokyo | 1 | 0 | 3 | 1 | 1 | 1 | 1 | 1 | 2 | 2 | 1 | 0 | 1 | 1 | 1 | 3 |
| Kanagawa | 1 | 1 | 3 | 1 | 1 | 1 | 1 | 1 | 2 | 3 | 2 | 1 | 1 | 1 | 1 | 3 |
| *Chubu* |  |  |  |  |  |  |  |  |  |  |  |  |  |  |  |  |
| Niigata | 1 | 1 | 3 | 1 | 2 | 3 | 1 | 1 | 2 | 2 | 2 | 1 | 1 | 1 | 1 | 1 |
| Toyama | 1 | 0 | 3 | 1 | 1 | 1 | 1 | 2 | 1 | 3 | 1 | 0 | 2 | 3 | 1 | 1 |
| Ishikawa | 1 | 1 | 3 | 1 | 1 | 2 | 1 | 2 | 3 | 1 | 2 | 1 | 2 | 1 | 1 | 1 |
| Fukui | 1 | 1 | 3 | 1 | 1 | 1 | 1 | 1 | 2 | 2 | 2 | 1 | 1 | 3 | 1 | 2 |
| Yamanashi | 1 | 1 | 2 | 1 | 1 | 1 | 1 | 1 | 1 | 2 | 2 | 1 | 1 | 2 | 2 | 1 |
| Nagano | 1 | 1 | 2 | 1 | 1 | 1 | 1 | 1 | 1 | 2 | 2 | 1 | 1 | 1 | 2 | 1 |
| Gifu | 1 | 0 | 2 | 1 | 1 | 1 | 1 | 1 | 1 | 2 | 1 | 0 | 1 | 1 | 1 | 1 |
| Shizuoka | 1 | 0 | 2 | 1 | 1 | 1 | 1 | 1 | 2 | 2 | 1 | 0 | 1 | 1 | 1 | 1 |
| Aichi | 1 | 1 | 3 | 1 | 1 | 1 | 1 | 1 | 1 | 3 | 1 | 1 | 1 | 2 | 1 | 1 |
| Mie | 1 | 1 | 2 | 1 | 2 | 1 | 1 | 1 | 1 | 1 | 1 | 1 | 1 | 1 | 1 | 1 |
| *Kinki* |  |  |  |  |  |  |  |  |  |  |  |  |  |  |  |  |
| Shiga | 1 | 1 | 2 | 1 | 1 | 1 | 1 | 1 | 2 | 2 | 1 | 1 | 1 | 1 | 1 | 1 |
| Kyoto | 1 | 1 | 2 | 1 | 1 | 1 | 1 | 2 | 1 | 2 | 1 | 1 | 2 | 2 | 1 | 2 |
| Osaka | 1 | 0 | 2 | 1 | 1 | 1 | 1 | 1 | 1 | 2 | 1 | 0 | 1 | 1 | 1 | 1 |
| Hyogo | 1 | 1 | 2 | 1 | 1 | 1 | 1 | 1 | 1 | 1 | 2 | 1 | 1 | 1 | 2 | 1 |
| Nara | 1 | 1 | 3 | 1 | 1 | 1 | 1 | 1 | 1 | 1 | 3 | 2 | 1 | 1 | 1 | 2 |
| Wakayama | 1 | 1 | 2 | 1 | 1 | 1 | 1 | 1 | 1 | 2 | 2 | 1 | 1 | 1 | 2 | 1 |
| *Chugoku* |  |  |  |  |  |  |  |  |  |  |  |  |  |  |  |  |
| Tottori | 1 | 0 | 3 | 1 | 1 | 1 | 1 | 1 | 2 | 3 | 1 | 0 | 1 | 1 | 1 | 1 |
| Shimane | 1 | 0 | 2 | 1 | 2 | 1 | 1 | 1 | 1 | 2 | 1 | 0 | 1 | 1 | 2 | 2 |
| Okayama | 1 | 1 | 2 | 1 | 1 | 1 | 1 | 1 | 1 | 2 | 1 | 2 | 1 | 2 | 1 | 1 |
| Hiroshima | 1 | 1 | 2 | 1 | 1 | 1 | 1 | 1 | 2 | 1 | 2 | 1 | 2 | 2 | 1 | 1 |
| Yamaguchi | 1 | 1 | 2 | 1 | 1 | 1 | 1 | 2 | 1 | 1 | 1 | 1 | 1 | 1 | 1 | 1 |
| *Shikoku* |  |  |  |  |  |  |  |  |  |  |  |  |  |  |  |  |
| Tokushima | 1 | 1 | 2 | 1 | 1 | 1 | 1 | 1 | 1 | 2 | 2 | 1 | 1 | 1 | 1 | 1 |
| Kagawa | 1 | 1 | 3 | 1 | 1 | 1 | 1 | 1 | 2 | 3 | 3 | 1 | 1 | 1 | 2 | 1 |
| Ehime | 1 | 0 | 3 | 2 | 1 | 1 | 1 | 1 | 3 | 2 | 1 | 0 | 1 | 2 | 3 | 3 |
| Kochi | 1 | 1 | 3 | 1 | 1 | 1 | 1 | 1 | 1 | 2 | 2 | 1 | 1 | 1 | 3 | 1 |
| *Kyushu* |  |  |  |  |  |  |  |  |  |  |  |  |  |  |  |  |
| Fukuoka | 1 | 0 | 2 | 2 | 1 | 1 | 1 | 1 | 1 | 2 | 1 | 0 | 2 | 2 | 1 | 1 |
| Saga | 1 | 0 | 2 | 2 | 1 | 1 | 1 | 1 | 1 | 2 | 1 | 0 | 1 | 2 | 1 | 1 |
| Nagasaki* | 1 | 0 | 3 | 1 | 1 | 1 | 1 | 2 | 3 | 2 | 1 | 0 | 1 | 3 | 1 | 1 |
| Kumamoto* | 1 | 0 | 3 | 1 | 1 | 1 | 1 | 1 | 3 | 1 | 1 | 0 | 1 | 2 | 1 | 1 |
| Oita | 1 | 0 | 3 | 1 | 1 | 1 | 2 | 1 | 1 | 3 | 1 | 0 | 1 | 2 | 1 | 1 |
| Miyazaki* | 1 | 0 | 2 | 2 | 2 | 1 | 1 | 2 | 1 | 1 | 1 | 0 | 2 | 1 | 2 | 1 |
| Kagoshima** | 2 | 1 | 3 | 2 | 2 | 2 | 3 | 2 | 2 | 2 | 2 | 1 | 1 | 3 | 1 | 2 |
| Okinawa | 2 | 1 | 3 | 2 | 2 | 2 | 3 | 2 | 2 | 1 | 1 | 1 | 2 | 2 | 1 | 1 |

*Time window is from week 18 to week 17 of the following year by the 2019/2020 season, then from week 45 to week 44 of the following year thereafter

**Time window is from week 18 to week 17 of the following year by the 2022/2023 season, then from week 1 to 52 thereafter.

Supplementary Table 5. Variability in cumulative cases per sentinel

| **National/prefecture** | **Mean (SD)** | **Median (Q1, Q3)** | **2012 / 2013** | **2013 / 2014** | **2014 / 2015** | **2015 / 2016** | **2016 / 2017** | **2017 / 2018** | **2018 / 2019** | **2019 / 2020** | **2020** | **2021** | **2022** | **2023** | **2024** |
| --- | --- | --- | --- | --- | --- | --- | --- | --- | --- | --- | --- | --- | --- | --- | --- |
| All | 37.4 (13.9) | 37.4 (30.9, 39.0) | 28.49 | 30.59 | 33.74 | 36.17 | 30.94 | 46.15 | 38.97 | 40.27 | 5.74 | 71.66 | 38.01 | 46.32 | 38.89 |
| Hokkaido | 43.9 (15.6) | 43.2 (36.6, 47.2) | 32.48 | 39.52 | 42.05 | 43.24 | 37.05 | 53.21 | 43.64 | 47.24 | 6.89 | 72.10 | 35.44 | 67.68 | 50.47 |
| *Tohoku* |  |  |  |  |  |  |  |  |  |  |  |  |  |  |  |
| Aomori | 23.2 (6.4) | 23.9 (22.0, 25.7) | 25.71 | 23.93 | 20.60 | 26.90 | 24.24 | 30.62 | 22.53 | 26.01 | 4.52 | 31.76 | 18.39 | 23.13 | 23.85 |
| Iwate | 34.1 (9.9) | 34.1 (27.0, 39.5) | 26.33 | 29.93 | 27.18 | 38.53 | 32.03 | 41.42 | 37.31 | 40.72 | 11.37 | 53.08 | 25.86 | 40.17 | 39.48 |
| Miyagi | 40.2 (19.7) | 40.2 (29.7, 44.3) | 16.48 | 23.81 | 36.10 | 42.34 | 35.86 | 50.59 | 41.66 | 44.87 | 7.47 | 91.45 | 31.64 | 56.04 | 44.30 |
| Akita | 22.9 (10.3) | 22.9 (18.0, 25.1) | 26.17 | 13.62 | 19.33 | 25.09 | 22.37 | 30.18 | 24.35 | 29.38 | 1.67 | 48.08 | 23.17 | 20.52 | 14.05 |
| Yamagata | 55.3 (22.2) | 55.3 (40.7, 63.7) | 35.88 | 40.98 | 39.93 | 61.70 | 56.23 | 67.65 | 54.45 | 63.69 | 4.44 | 105.01 | 54.28 | 69.51 | 64.80 |
| Fukushima | 56.8 (19.9) | 56.8 (49.4, 67.1) | 45.44 | 53.28 | 56.85 | 67.63 | 54.41 | 71.84 | 58.58 | 67.12 | 7.16 | 95.20 | 38.18 | 71.75 | 50.78 |
| *Kanto* |  |  |  |  |  |  |  |  |  |  |  |  |  |  |  |
| Ibaraki | 22.3 (7.4) | 23.8 (19.7, 24.6) | 15.52 | 18.20 | 23.83 | 24.24 | 20.17 | 29.87 | 25.26 | 26.42 | 2.40 | 34.52 | 23.99 | 24.57 | 20.62 |
| Tochigi | 32.7 (10.2) | 32.7 (28.6, 37.9) | 26.64 | 28.71 | 30.65 | 35.77 | 28.17 | 38.12 | 32.54 | 37.91 | 3.50 | 48.42 | 39.75 | 41.09 | 33.50 |
| Gunma | 26.3 (8.7) | 25.4 (24.5, 27.3) | 22.77 | 24.33 | 27.32 | 24.61 | 24.90 | 34.86 | 29.21 | 32.16 | 2.13 | 42.71 | 25.43 | 26.89 | 25.02 |
| Saitama | 29.3 (11.4) | 29.1 (24.4, 30.1) | 19.99 | 24.46 | 28.74 | 25.93 | 24.18 | 39.46 | 30.12 | 35.12 | 3.49 | 56.03 | 29.09 | 34.87 | 29.84 |
| Chiba | 22.1 (10.0) | 21.0 (17.2, 24.2) | 15.72 | 16.74 | 17.64 | 17.37 | 20.19 | 27.80 | 22.90 | 24.19 | 2.39 | 48.92 | 26.28 | 25.84 | 20.97 |
| Tokyo | 28.3 (12.6) | 26.3 (24.2, 29.1) | 23.20 | 22.91 | 25.30 | 26.27 | 25.88 | 36.61 | 30.33 | 31.56 | 2.18 | 63.01 | 29.12 | 27.01 | 24.52 |
| Kanagawa | 21.4 (10.6) | 21.0 (16.6, 23.8) | 11.87 | 14.23 | 17.50 | 17.35 | 18.46 | 30.24 | 23.39 | 23.84 | 2.37 | 49.83 | 23.99 | 24.20 | 21.00 |
| *Chubu* |  |  |  |  |  |  |  |  |  |  |  |  |  |  |  |
| Niigata | 57.9 (28.3) | 57.9 (46.2, 63.8) | 46.03 | 46.90 | 46.23 | 48.35 | 59.88 | 76.17 | 63.84 | 77.62 | 7.28 | 129.10 | 21.03 | 71.49 | 58.24 |
| Toyama | 42.6 (23.2) | 42.1 (31.9, 44.7) | 25.00 | 23.00 | 34.24 | 37.98 | 42.14 | 49.18 | 43.82 | 36.01 | 2.79 | 107.71 | 44.68 | 59.39 | 47.41 |
| Ishikawa | 42.0 (25.4) | 36.9 (29.8, 42.0) | 29.66 | 26.31 | 36.90 | 36.41 | 39.62 | 55.59 | 45.59 | 41.62 | 3.72 | 119.53 | 34.01 | 47.43 | 29.85 |
| Fukui | 52.2 (25.6) | 46.3 (40.5, 54.4) | 42.14 | 40.36 | 34.86 | 54.41 | 40.59 | 63.59 | 50.27 | 44.96 | 4.52 | 121.98 | 46.29 | 61.74 | 72.28 |
| Yamanashi | 20.3 (8.3) | 21.3 (15.0, 24.4) | 7.00 | 14.17 | 15.33 | 22.42 | 21.04 | 28.19 | 24.43 | 22.74 | 1.75 | 32.50 | 25.23 | 27.91 | 21.31 |
| Nagano | 30.1 (11.2) | 27.3 (24.7, 32.2) | 24.44 | 25.56 | 32.21 | 41.71 | 24.78 | 34.51 | 27.29 | 31.46 | 6.50 | 57.09 | 23.37 | 35.73 | 26.86 |
| Gifu | 31.1 (14.1) | 27.3 (22.8, 32.9) | 22.08 | 23.06 | 27.30 | 30.10 | 19.80 | 33.63 | 26.67 | 27.11 | 3.45 | 60.46 | 48.34 | 49.52 | 32.91 |
| Shizuoka | 35.1 (16.9) | 31.8 (29.8, 36.3) | 30.02 | 30.12 | 33.98 | 29.13 | 23.65 | 40.83 | 31.17 | 36.27 | 3.03 | 82.96 | 31.81 | 46.36 | 37.19 |
| Aichi | 28.0 (10.2) | 27.7 (23.7, 29.7) | 21.79 | 24.04 | 24.78 | 27.68 | 22.82 | 33.58 | 29.72 | 29.32 | 3.36 | 48.21 | 41.79 | 32.56 | 24.98 |
| Mie | 50.8 (26.2) | 45.0 (34.9, 55.3) | 27.56 | 30.69 | 38.44 | 44.76 | 36.33 | 55.32 | 57.51 | 45.01 | 6.57 | 114.71 | 86.18 | 65.10 | 52.11 |
| *Kinki* |  |  |  |  |  |  |  |  |  |  |  |  |  |  |  |
| Shiga | 26.0 (8.5) | 26.0 (24.6, 27.8) | 31.38 | 25.22 | 26.25 | 27.77 | 18.11 | 25.54 | 22.94 | 25.86 | 2.63 | 38.03 | 27.83 | 37.16 | 29.64 |
| Kyoto | 24.2 (10.3) | 24.2 (17.3, 27.8) | 17.28 | 15.88 | 20.58 | 20.96 | 17.30 | 27.83 | 24.84 | 25.68 | 2.29 | 46.13 | 35.93 | 32.24 | 27.90 |
| Osaka | 48.9 (16.6) | 48.9 (41.9, 54.2) | 36.32 | 38.53 | 47.14 | 46.46 | 43.03 | 59.35 | 56.47 | 52.75 | 5.84 | 81.41 | 60.72 | 53.78 | 54.20 |
| Hyogo | 41.4 (17.8) | 40.5 (30.2, 44.9) | 27.34 | 30.07 | 36.81 | 35.35 | 30.19 | 50.76 | 40.45 | 44.27 | 5.01 | 82.94 | 50.31 | 60.17 | 44.92 |
| Nara | 46.3 (20.2) | 49.3 (31.9, 54.1) | 21.29 | 26.67 | 33.62 | 50.65 | 43.59 | 60.13 | 51.41 | 54.15 | 5.12 | 89.28 | 49.26 | 58.34 | 58.55 |
| Wakayama | 44.6 (21.3) | 37.5 (33.7, 44.8) | 24.44 | 32.91 | 37.00 | 34.17 | 33.97 | 44.78 | 55.48 | 43.03 | 10.54 | 98.42 | 37.47 | 71.11 | 56.62 |
| *Chugoku* |  |  |  |  |  |  |  |  |  |  |  |  |  |  |  |
| Tottori | 43.2 (15.4) | 43.2 (37.8, 48.6) | 32.37 | 43.89 | 44.53 | 57.63 | 38.42 | 50.02 | 35.89 | 41.87 | 2.62 | 73.73 | 48.64 | 48.59 | 43.10 |
| Shimane | 44.1 (15.4) | 43.0 (39.5, 44.9) | 42.96 | 39.68 | 44.86 | 54.61 | 38.91 | 53.15 | 42.02 | 40.26 | 3.41 | 70.38 | 44.11 | 64.06 | 35.02 |
| Okayama | 25.0 (8.5) | 24.6 (20.9, 28.3) | 19.61 | 18.84 | 23.09 | 23.78 | 21.30 | 30.27 | 25.87 | 28.33 | 3.37 | 39.49 | 24.60 | 33.98 | 31.99 |
| Hiroshima | 39.5 (12.8) | 39.5 (35.7, 42.9) | 35.92 | 34.90 | 44.46 | 41.11 | 31.10 | 48.47 | 37.75 | 42.86 | 4.43 | 63.43 | 41.42 | 49.01 | 38.26 |
| Yamaguchi | 64.2 (25.2) | 63.0 (52.3, 66.4) | 53.43 | 63.04 | 48.77 | 65.51 | 44.23 | 67.62 | 66.44 | 62.88 | 12.72 | 130.13 | 62.41 | 84.69 | 72.46 |
| *Shikoku* |  |  |  |  |  |  |  |  |  |  |  |  |  |  |  |
| Tokushima | 69.9 (26.7) | 69.9 (55.4, 78.5) | 56.31 | 77.81 | 84.13 | 78.48 | 72.04 | 90.79 | 85.09 | 60.68 | 6.04 | 126.65 | 52.48 | 69.21 | 49.59 |
| Kagawa | 45.2 (15.5) | 44.8 (40.0, 49.4) | 44.80 | 44.47 | 44.35 | 55.86 | 33.75 | 53.08 | 49.35 | 60.27 | 4.78 | 74.34 | 36.18 | 45.14 | 41.21 |
| Ehime | 53.3 (19.7) | 53.3 (42.6, 62.0) | 43.59 | 39.65 | 50.03 | 55.38 | 36.57 | 68.07 | 55.17 | 52.56 | 3.08 | 85.08 | 61.97 | 72.63 | 69.26 |
| Kochi | 44.9 (24.0) | 40.2 (33.7, 45.1) | 33.87 | 33.23 | 36.03 | 45.10 | 27.77 | 58.76 | 43.08 | 38.03 | 5.93 | 114.70 | 40.21 | 48.91 | 58.16 |
| *Kyushu* |  |  |  |  |  |  |  |  |  |  |  |  |  |  |  |
| Fukuoka | 56.4 (22.2) | 54.7 (49.1, 56.4) | 52.99 | 49.94 | 46.54 | 54.70 | 43.03 | 72.60 | 54.96 | 55.42 | 5.95 | 110.80 | 54.62 | 73.48 | 58.73 |
| Saga | 51.3 (24.5) | 46.5 (41.6, 51.7) | 43.83 | 42.09 | 42.09 | 40.17 | 33.63 | 46.47 | 54.64 | 51.72 | 6.10 | 117.86 | 50.89 | 71.48 | 65.71 |
| Nagasaki* | 39.8 (16.4) | 38.1 (32.0, 46.1) | 29.93 | 34.32 | 37.45 | 32.75 | 23.20 | 54.86 | 38.09 | 46.08 | 2.79 | 72.58 | 39.72 | 53.18 | 52.06 |
| Kumamoto* | 44.9 (17.6) | 41.8 (35.1, 49.8) | 35.28 | 41.80 | 51.98 | 41.25 | 38.56 | 68.60 | 49.26 | 49.80 | 4.30 | 79.22 | 34.55 | 56.83 | 32.64 |
| Oita | 42.8 (19.8) | 39.8 (32.2, 42.8) | 35.25 | 27.64 | 30.89 | 41.00 | 32.64 | 39.82 | 40.47 | 38.09 | 3.44 | 78.49 | 56.81 | 80.40 | 51.02 |
| Miyazaki* | 65.3 (23.3) | 65.9 (62.2, 70.6) | 70.58 | 62.47 | 67.50 | 61.28 | 47.58 | 71.71 | 65.90 | 77.92 | 3.26 | 115.55 | 64.22 | 74.08 | 67.43 |
| Kagoshima** | 55.2 (17.2) | 52.4 (37.9, 62.4) | 33.96 | 46.35 | 37.75 | 46.25 | 34.20 | 62.35 | 57.83 | 52.39 | 68.81 | 87.46 | 37.89 | 81.25 | 70.54 |
| Okinawa | 51.1 (12.3) | 48.9 (45.0, 58.6) | 25.79 | 47.06 | 40.97 | 58.62 | 46.38 | 69.20 | 60.50 | 71.41 | 46.31 | 60.81 | 38.08 | 49.75 | 48.87 |

*Time window is from week 18 to week 17 of the following year by the 2019/2020 season, then from week 45 to week 44 of the following year thereafter

**Time window is from week 18 to week 17 of the following year by the 2022/2023 season, then from week 1 to 52 thereafter.
